# Loss of Cyclin C or CDK8 provides ATR inhibitor resistance by suppressing transcription-associated replication stress

**DOI:** 10.1093/nar/gkab628

**Published:** 2021-07-30

**Authors:** Rebecca L Lloyd, Vaclav Urban, Francisco Muñoz-Martínez, Iñigo Ayestaran, John C Thomas, Christelle de Renty, Mark J O’Connor, Josep V Forment, Yaron Galanty, Stephen P Jackson

**Affiliations:** Wellcome/Cancer Research UK Gurdon Institute, and Department of Biochemistry, University of Cambridge, UK; Bioscience, Oncology R&D, AstraZeneca, Cambridge, UK; Wellcome/Cancer Research UK Gurdon Institute, and Department of Biochemistry, University of Cambridge, UK; Wellcome/Cancer Research UK Gurdon Institute, and Department of Biochemistry, University of Cambridge, UK; Wellcome/Cancer Research UK Gurdon Institute, and Department of Biochemistry, University of Cambridge, UK; Bioscience, Oncology R&D, AstraZeneca, Cambridge, UK; Bioscience, Oncology R&D, AstraZeneca, Cambridge, UK; Bioscience, Oncology R&D, AstraZeneca, Cambridge, UK; Wellcome/Cancer Research UK Gurdon Institute, and Department of Biochemistry, University of Cambridge, UK; Wellcome/Cancer Research UK Gurdon Institute, and Department of Biochemistry, University of Cambridge, UK

## Abstract

The protein kinase ATR plays pivotal roles in DNA repair, cell cycle checkpoint engagement and DNA replication. Consequently, ATR inhibitors (ATRi) are in clinical development for the treatment of cancers, including tumours harbouring mutations in the related kinase ATM. However, it still remains unclear which functions and pathways dominate long-term ATRi efficacy, and how these vary between clinically relevant genetic backgrounds. Elucidating common and genetic-background specific mechanisms of ATRi efficacy could therefore assist in patient stratification and pre-empting drug resistance. Here, we use CRISPR–Cas9 genome-wide screening in ATM-deficient and proficient mouse embryonic stem cells to interrogate cell fitness following treatment with the ATRi, ceralasertib. We identify factors that enhance or suppress ATRi efficacy, with a subset of these requiring intact ATM signalling. Strikingly, two of the strongest resistance-gene hits in both ATM-proficient and ATM-deficient cells encode Cyclin C and CDK8: members of the CDK8 kinase module for the RNA polymerase II mediator complex. We show that Cyclin C/CDK8 loss reduces S-phase DNA:RNA hybrid formation, transcription-replication stress, and ultimately micronuclei formation induced by ATRi. Overall, our work identifies novel biomarkers of ATRi efficacy in ATM-proficient and ATM-deficient cells, and highlights transcription-associated replication stress as a predominant driver of ATRi-induced cell death.

## INTRODUCTION

Ataxia telangiectasia and Rad3-related (ATR) is a fundamental DNA damage response (DDR) protein kinase involved in DNA double-strand break (DSB) signalling and cell cycle checkpoint engagement, and is also an apical regulator of the replication stress response (RSR) (1–3). ATR’s roles in the stabilisation and restart of stalled DNA replication forks are important for maintaining genomic integrity since, in the absence of ATR function, stalled replication forks are converted into cytotoxic DSBs through replication fork collapse. ATR also plays pivotal roles in unperturbed S-phase by regulating dormant replication-origin firing and controlling the S/G2 cell cycle transition (4). Importantly, replication stress (5) has been identified as a hallmark of cancer (6). While this may be in-part due to faster proliferation rates and nucleotide shortages in S-phase, amplification of the oncogenes *CCNE1* and *MYC* specifically induce replication stress by shortening G1 and promoting firing of intragenic origins that would otherwise be repressed by near-completed transcription (7). This also increases conflicts between DNA replication and transcription (8), with such collisions causing genome instability through replication fork collapse (9,10). Replication stress, particularly in the absence of replication fork protection, can also lead to exhaustion of replication protein A (RPA). This results in insufficient RPA to coat the single-stranded DNA (ssDNA) that arises during replication fork stalling or the uncoupling of the DNA helicase and DNA polymerase, ultimately leading to global replication fork collapse and replication catastrophe (11). Importantly, ATR’s fundamental roles in the RSR, including limiting origin firing and promoting nucleotide synthesis, act to prevent RPA exhaustion and replication catastrophe, and likely present a key survival mechanism for cancer cells with high endogenous replication stress.

Exploiting this concept, ATR inhibitors (ATRi) are in clinical development for the treatment of cancers (12) including those with mutations in the related kinase ataxia telangiectasia mutated (ATM) (13–16), or high endogenous replication stress (17–20). The latter is also a biomarker for hypersensitivity towards inhibitors of CHK1 and WEE1 kinases, both of which also play key roles in the RSR (17). Recent studies using CRISPR–Cas9 and siRNA screening methodologies identified loss of POLE3/4, RNASEH2A/B, and ERCC1 as additional biomarkers of ATRi hypersensitivity (21–23). Similarly, loss of the cyclin-dependent kinase (CDK)-regulator phosphatase CDC25A was found to promote ATRi resistance, implicating abrogation of the G2/M checkpoint as a major driver of ATRi efficacy (24). However, these studies have thus far been performed in wild-type (WT) or *TP53* knockout cells to facilitate screen performance. This may mean that identified hits do not fully reflect the drivers of cell death in tumours harbouring specific genetic defects, such as mutations in *ATM*, that are likely to be targeted with ATR inhibitors in the clinic. In particular, the hypersensitivity of *ATM*-mutated cancers to ATR inhibition (13–16) could be driven by over-lapping and redundant functions of ATM and ATR kinases, given that there are over 700 suggested ATM/ATR substrates harbouring S/T-Q motifs (25–27). ATM and ATR signalling are already known to crosstalk to regulate processes including DNA repair, cell senescence and apoptosis, and cell cycle checkpoint control (28–31), with roles for ATM in regulating DNA replication having also been reported (32–35). It is therefore likely that drivers of ATRi sensitivity and resistance will differ between ATM-proficient and ATM-deficient cells, meaning that elucidating both common and genetic background-specific mechanisms of ATRi efficacy will be critical for accurately stratifying patients and pre-empting innate or acquired drug resistance.

To address the above issues, in this study we examine genetic drivers of ATRi efficacy by performing pooled CRISPR–Cas9 screens in *Atm* WT and *Atm* knockout (KO) mouse embryonic stem cells (mESCs) using the ATRi, ceralasertib (AZD6738) (36). Our ensuing data reveal how drivers of ATRi efficacy depend on functional ATM expression, thereby providing further insight into how ATR and ATM cooperate to maintain genomic integrity. Furthermore, by focusing on the strongest resistance-gene hits in both ATM-proficient and ATM-deficient cells, we investigate the mechanism by which loss of Cyclin C or CDK8, members of the CDK8 kinase module of the RNA polymerase II (RNAPII) mediator complex, promote resistance to ATR and CHK1 inhibition by limiting DNA:RNA hybrid formation in S-phase and transcription-associated replication stress. These findings also suggest new therapeutic opportunities for ATR inhibitors and provide insights for better stratifying patients and pre-empting innate or acquired drug resistance.

## MATERIALS AND METHODS

### Cell lines and compounds

Cell line origins and their cell growth media can be found in Supplementary Methods Table S1. mESC, FaDu and A549 *ATM*-knockout cell lines were generated as described before (37,38). mESC *Cdc25a* KO clones #4/5 were kindly provided by Oscar Fernández-Capetillo (24) and U2-OS T-Rex GFP-RNase H1(D210N) (RNH1(D210N)-GFP) or GFP-RNase H1 (RNH1-GFP) cells by Pavel Janscak (39). mESCs were cultured on 0.1% gelatin-coated tissue culture flasks, and Cas9-expressing cell lines were maintained in blasticidin (10 μg/ml mESC and U2-OS, 5 μg/ml HAP-1). AZD6738 and AZD1775 were made by AstraZeneca. DRB (5,6-dichloro-1-beta-ribo-fuanosyl benzimidazole), actinomycin D, aphidicolin, hydroxyurea, carboplatin and etoposide were obtained from Sigma-Aldrich. XL413 (S7547), VE-822 (S27102), LY2603618 (S2626) and LY2606368 (S7178) were obtained from SelleckChem. CX-5461 (509265) was obtained from Merck Millipore, and BRD-6989 (6438) from Tocris. Final assay DMSO concentrations were normalised to 1:1000 as required. Recombinant human IFNγ was obtained from Peprotech (300–02) and used at a final concentration of 2.5 ng/ml.

### *In vitro* growth and cell viability assays

#### MTT cell proliferation assays

For each biological replicate (*n* = 3) cells were seeded as technical replicates at 5000 cells per well of a 96-well plate, 16 h prior to drug treatment. Following treatment, medium was replaced with 50 μl 0.5 mg/ml MTT (3-[4,5-dimethylthiazol-2-yl]-2,5- diphenyltetrazolium bromide; thiazolyl blue) in growth media and cells incubated for a further 4 h. 50 μl 10% SDS was then added to the cells and incubated at 37°C overnight before reading absorbance at 595 nm.

#### Clonogenic survival assays

For each biological replicate (*n* = 3), 16 h prior to treatment cells were seeded in triplicate in 6-well plates at 500 cells/well (mESC, A549, U2-OS, HAP-1) or 1500 cells/well (FaDu). *ATM* KO FaDu cells were seeded in conditioned media. Cells were incubated with the drug for 6 (mESC, HAP-1) or 9 days (A549, FaDu, U2-OS) before methanol fixation (mESC) and crystal violet staining. The number of colonies (defined as containing > 30 cells) were counted blind and normalised to the untreated conditions to account for variations in plating efficiencies. For short-term treatments, cells were washed three times with fresh drug-free media following treatment.

#### Incucyte cell confluency

For each biological replicate, cells were seeded as technical replicates at 5000 cells per well of a 96-well plate. Live-cell imaging was acquired at 10× magnification every 2 h, and percentage phase confluency was quantified using Incucyte ZOOM 2018A software (Essen Bioscience). Doubling times were calculated in GraphPad prism V.8 during the exponential growth phase using the exponential growth equation.

#### CRISPR–Cas9 screen for resistance and sensitivity to AZD6738

1 × 10^8^*Atm* WT or KO mESCs were independently infected with a pre-packaged lentiviral library at a MOI (multiplicity of infection) of 0.1, giving a library coverage of ∼1000×. The Kosuke Yusa murine v2 sgRNA library was used (40). After 48 h, 1 × 10^8^ cells/genotype were collected for sequencing and a further 1 × 10^8^ cells treated with puromycin (2 μg/ml) for 12 days to select for cells with the stably integrated sgRNA cassette. Following establishment of puromycin resistance (day 6) cells were partitioned into three technical replicates and at least 5 × 10^7^ cells (500×) per sample were maintained in culture for the remainder of the screen. A total puromycin selection period of 12 days ensured that even slow-turnover proteins were depleted and allowed technical replicates to become established as independent samples. On day 14, 5 × 10^7^ cells/replicate were collected for sequencing and 5 × 10^7^ cells/replicate plated for each genotype and treatment condition. Sixteen hours later, cells were treated with either DMSO, IC_10_ AZD6738 (100 nM *Atm* KO; 350 nM WT) or IC_90_ AZD6738 (350 nM *Atm* KO; 900 nM WT) for 6 days and then replated in drug-free medium for 48 h recovery. A 6-day treatment period was chosen to allow the controlled induction of ATRi-driven cell death through multiple rounds of cell division, using ATR-selective concentrations of AZD6738, while minimising the time for genetic drift to occur. IC_10_ and IC_90_ are the inhibitory doses that result in a 10% or 90% reduction in cell number respectively when assessed in screening format. On days 2 and 5 of treatment (every 3 days), cells were passaged and 5 × 10^7^ cells per treatment condition were re-seeded to ensure that cells were maintained in optimal conditions and below confluency throughout the screen. Surviving cells were separately pooled for each condition and replicate and pelleted for DNA extraction and next-generation sequencing. DNA was extracted at 55°C in TAIL buffer (17 mM Tris pH 7.5, 17 mM EDTA, 170 mM NaCl, 0.85% SDS, 1 mg/ml proteinase K), isopropanol precipitated, purified in 70% ethanol, and dissolved to 200 ng/μl in H_2_O. DNA was amplified by PCR (2 μg × 96 reactions, or 960 reactions for 48-hour samples; approximately 319× representation) using Q5 high fidelity polymerase and extended primers containing Illumina adaptors and barcodes for multiplexing. To reduce PCR bias, the number of cycles was limited to 26, and multiple PCR reactions were performed in parallel. The product was cleaned using SPRIselect beads (Beckman Coulter, B23317) and column purification (Qiagen, 28106), and all samples of the same genotype multiplexed using q-PCR NEBNext library quant kit (E7630). The 19 bp sgRNA (along with 1 bp on either side) and 8 bp barcode were sequenced on an Illumina Hiseq1500 system using single-ended reads. An in-house script was used to count the number of each sgRNA per pool, and enriched or depleted genes determined by comparison of control and treated samples using the software package MAGeCK (0.5.5). WT IC_10_ technical replicate 1 was excluded from the analyses due to principal component analysis (PCA) indicating that this sample closely resembled the DMSO-treated samples. Given the data represent technical replicates, data were independently analysed by both summing the replicates, and by using the MAGeCK replicate function. Due to noise in low read count essential genes sometimes confounding drug-treatment results, sgRNAs with low read counts in the DMSO control were excluded prior to analysis (41). No sgRNAs were excluded for essential gene comparisons between 48 h and day 14 (pre-treatment) samples. For summed analyses, sgRNAs with DMSO read counts <30 after combining the replicates were excluded (SUM < 30ex). For replicates analyses, sgRNAs with DMSO read counts of 0 in any 2 replicates and <10 in the third were excluded (REP < 10ex). Drop-out and enrichment hits were determined using each method at both IC_10_ and IC_90_ doses (Supplementary Table S1), and the analyses which provided the greatest number of significant hits (false discovery rate (FDR) < 0.1) are visualised in the main figure panels. The most significant hits were determined to have a FDR of <0.1, but hits with a *P*-value <0.001 were also assessed as potential candidates.

Format of MAGeCK commands used:

Summed analysis: mageck test -k counts.csv -c DMSO -t ATRi -n results.csv

Replicates analysis: mageck test -k counts.csv -c ‘DMSO1’,‘DMSO2’,‘DMSO3’ -t ‘ATRi1’,‘ATRi2’,‘ATRi3’ -n results.csv

Essential gene analysis: mageck test -k counts.csv -c 48h -t D14 -n results.csv

#### Flow cytometry

Cells were seeded at 500 000 cells/well in 6-well plates 18 h prior to treatment. Cells were incubated with compounds and EdU (5-ethynyl-2′-deoxyuridine; 10 μM; Sigma-Aldrich 900584) or EU (5-ethynyl uridine; 1 mM; ab146642) as indicated and fixed in 70% ice-cold ethanol. Antibody stainings were performed in 1 mg/ml BSA-PBS as required using the antibodies listed in Supplementary Methods Table S2. The click reactions were subsequently performed as previously reported (42) before staining with 1 μg/ml DAPI in PBS containing 250 μg/ml RNase A. All samples were acquired using Aurora (Cytek Biosciences), Fortessa (BD biosciences) or Cytoflex (Beckman Coulter) flow cytometers, and analysed in FlowJo (Tree Star).

#### Western blot analyses

Cells were lysed in 50 mM Tris pH 7.5, 2% SDS, 10 mM N-ethylmaleimide supplemented with protease inhibitors (Roche) and phosphatase inhibitors (Sigma-Aldrich), and heated to 95°C for 5 min. For chromatin fractionations, cells were lysed in CSK buffer (10 mM PIPES, 100 mM NaCl, 300 mM sucrose, 3 mM MgCl_2_, 0.7% Triton X-100, supplemented with protease and phosphatase inhibitors) for 30 min on ice and centrifuged for 10 min max speed at 4°C, with the supernatant removed as the soluble fraction. The pellet was washed twice in ice-cold PBS, resuspended in CSK buffer and syringed to produce the chromatin fraction. Equal amounts of lysates were separated on 4–12% Bis-Tris NuPAGE gels and analysed by standard immunoblotting using the antibodies listed in Supplementary Methods Table S2.

#### Immuno-fluorescence

Cells were plated in 96-well plates (20 000 cells/well U2-OS, 50 000 cells/well HAP-1) 16 h prior to treatment. For U2-OS RNH1-GFP cells, 12 000 cells/well were plated and induced 16 h later with 1 ng/ml doxycycline for 24 h prior to treatment. Cells were incubated with compounds, EdU (10 μM) or EU (1 mM) as indicated, and fixed in 4% PFA for 15 min at room temperature (RT). For γH2AX and RPA32 staining, cells were pre-extracted in ice-cold 0.2% Triton-PBS for 10 min on ice prior to fixation. Cells were permeabilised in 0.5% Triton-PBS for 15 min and blocked in 5% BSA in 0.1% PBS–tween (PBST). As required, the click reactions were performed as previously reported (42) before antibody staining. For antibody staining, primary antibodies were incubated at 4°C O/N using the antibodies listed in Supplementary Methods Table S2. Cells were washed 3× in PBST before incubation with Alexa Fluor secondary antibodies for 1 h at RT in the dark, followed by DAPI staining (1 μg/ml) for 20 min at RT. Plates were imaged using an Opera Phenix spinning disc confocal microscope (PerkinElmer) at 40× magnification. All data were imported into Harmony image analysis software (PerkinElmer) for subsequent analyses.

#### Defining and counting nuclei

Nuclei were defined based on DAPI intensity. Border objects were removed from analyses.

#### Defining and counting micronuclei

Micronuclei were initially defined based upon the DAPI stain using Harmony's ‘find micronuclei’ function, and further refined based upon the following parameters: fraction of nucleus area <0.33, roundness >0.75, area >2 μm^2^, fraction of nucleus intensity >0.25, intensity DAPI CV (%) <36 and distance from nucleus 0.6–10 μm.

#### Calculating nuclear intensities

Mean intensities were calculated in pre-defined nuclei and used to define EdU positive nuclei for further analyses as required.

#### Counting nuclear foci

The ‘find spots’ function was used to identify foci in pre-defined nuclei, and foci were subsequently refined to exclude spots <1 px^2^.

#### Proximity ligation assays

The PLA assays were performed as previously described (43), with 20 000 cells/well plated in 96-well plates 16 h prior to treatment. Plates were imaged using an Opera Phenix spinning disc confocal microscope (PerkinElmer) at 40× magnification. All data were imported into Harmony image analysis software (PerkinElmer) for subsequent analyses.

#### DsiRNA transfections

DsiRNAs (27-mer Dicer-substrate short interfering RNAs) were obtained from IDT and transfected using Lipofectamine RNAiMAX (Invitrogen) according to manufacturer's protocol. The non-targeting siRNA (NTsi) NC was obtained from IDT and used as the control. Cells were plated for assays 48 h later. DsiRNA sequences can be found in Supplementary Methods Table S3.

#### RNH1(D210N)-GFP reporter assay (39)

RNH1(D210N)-GFP cells were transfected with the indicated DsiRNAs. Twenty-four hours post transfection, cells were seeded in 96 well plates in the presence of doxycycline (1 ng/ml) to induce expression of GFP-RNase H1(D210N) and treated with inhibitors after 24 h. For analyses of cell cycle distributions, EdU was added to cell culture medium at a final concentration of 10 μM for 30 min prior to fixation. Cells were pre-extracted (25 mM HEPES–NaOH pH 7.5, 50 mM NaCl, 1 mM EDTA, 3 mM MgCl_2_, 0.3 M sucrose and 0.5% Triton X-100) for 4 min on ice before fixation in 4% PFA. EdU click reactions were performed in buffer containing 100 mM Tris–HCl (pH 8.5), 2 mM CuSO_4_, 100 mM sodium ascorbate and 5 μM Alexa Fluor 647 azide (Thermo Fisher Scientific) for 20 min, and DNA was counterstained with 1 μg/ml DAPI. Image acquisition was performed on a WiScan Hermes system (IDEA Bio-Medical) equipped with 40×/0.75 NA air objective, and nuclear GFP foci counts were analysed using integrated WiSoft Athena software.

#### Stable cell line generation

Lentivirus-based CDK8-GFP and CDK8(D173A)-GFP plasmids were obtained from VectorBuilder, packaged in HEK293-lentiX cells using second generation packaging plasmids, and the lentivirus used to infect HAP-1 cells. Forty-eight hours later, cells with the integrated plasmids were selected in puromycin (0.5 μg/ml) for 7 days prior to single-cell plating and clonal expansion. For gene knockouts using CRISPR, synthetic crRNA (CRISPR RNA) and tracrRNAs (trans-activating RNA) were obtained from IDT (Supplementary Methods Table S4), duplexed and transfected into Cas9-expressing cells using RNAiMAX according to the manufacturer's instructions. Pooled editing efficiencies were assessed after 72 h and cells plated for single-cell expansion. Individual clones were tested for successful and complete KO via western blotting. All KO clones used in this study were genotype validated (Supplementary Table S2) by either TIDe (44) or TOPO-cloning (Thermofisher, K28002) following genomic DNA extraction and PCR amplification of the sgRNA-targeted loci (associated primers can be found in Supplementary Methods Table S4).

#### RT-qPCR

RNA was extracted using a RNeasy kit with on column DNase digestion according to the manufacturer's protocol (Qiagen, 74106). 3 μg total RNA (quantified using a NanoDrop spectrophotometer) was used for reverse transcription using SuperScriptIII first-strand synthesis reagents and oligo dT according to the manufacturer's protocol (Invitrogen, 18080051). cDNA was diluted in a final volume of 200 μl nuclease-free water and further diluted one in five prior to amplification, resulting in Ct values in the range of 20–30 cycles. Quantitative PCR was performed using 2× Fast SYBR Green Master mix (Thermo Fisher Scientific, 4385612) in a final volume of 20 μl, using 2 μl cDNA (post one in five dilution). For each biological replicate, individual amplification reactions were performed in technical triplicates, and primers targeting the house-keeping gene *Gapdh* were included in separate wells for each condition to normalise transcript levels. Primers were designed to span an exon-exon junction using primer-BLAST, and synthesised by Sigma-Aldrich. Sequences can be found in Supplementary Methods Table S5. The StepOne Plus Real Time PCR system (Thermo Fisher Scientific) was used to obtain raw Ct values, and the comparative Ct method 2^–ΔΔCt^ used to quantify transcript levels.

#### CRISPR-based cell competition assays

Synthetic crRNA and tracrRNAs were obtained from IDT (Supplementary Methods Table S4), duplexed and transfected into Cas9-expressing mESCs using RNAiMAX according to manufacturer's protocol. Cells were cultured for 7 days to allow gene editing and protein depletion to occur, resulting in a mixed population of edited and non-edited cells. 5 × 10^5^ cells were then plated per 10 cm dish and treated with either DMSO or 1.5 μM AZD6738 for 5 days, including passaging as required. 1.5 μM AZD6738 had been optimised to kill ∼95% of non-edited control cells in this assay format, thereby strongly enriching for resistant cells. A sgRNA targeting *Lrrc29* was used as a negative control as our screen outcomes did not predict this gene to influence ATRi sensitivity in *Atm* WT mESCs. Genomic DNA from surviving cells, alongside from non-transfected control cells, was extracted (Invitrogen, K1820) and the sgRNA-target loci PCR amplified using the associated primers in Supplementary Methods Table S4. The forward primer was used for Sanger sequencing of the PCR product. Sequence traces between transfected and non-transfected cells were compared using TIDe software (3.2.0) (44), and used to calculate the % of each InDel (–50 bp to + 50 bp) per sample. To account for in-frame mutations likely having minimal impact on protein expression, InDels were classified as in-frame or out-of-frame based on whether the size of the InDel was a multiple of 3. Undefined InDels could be the result of noise in the Sanger sequencing trace, or InDels outside the range –50 bp to +50 bp.

#### RNA-seq

RNA sequencing (RNA-seq) was performed in mESCs using two WT biological replicates and three independent *Ccnc* KO and *Cdk8* KO clones. Cells were treated for 4 h with either DMSO or 900 nM AZD6738 prior to sample collection. A 4-hour timepoint gave the biggest window in *Ccnb1* mRNA expression, which has been shown to be prematurely upregulated upon ATR inhibition (4) (data not shown). RNA was extracted using a RNeasy kit with on column DNase digestion according to the manufacturer's protocol (Qiagen, 74106). RNA integrity was confirmed using Agilent Tapestation RNA screentape, showing two sharp peaks representing the 18S and 28S ribosomal subunits, with a 28S:18S ratio of approximately 2 (RINe > 9). Using 5 μg input RNA, mRNA was isolated from ribosomal RNA using the NEBNext Poly(A) mRNA magnetic isolation module (E7490) according to the manufacturer's instructions. Following two rounds of enrichment, successful isolation and sample integrity (RINe 2–3) were confirmed using high sensitivity Agilent Screen tape. Library preparation was performed using the NEBNext Ultra II directional library prep kit (E7760) according to the manufacturer's instructions for ‘purified RNA or clean RNA’, and using NEBnext multiplex oligos (E7335). cDNA concentrations were measured using the Qubit dsDNA assay kit (Q32854), and molar concentrations calculated using the average DNA fragment size following analyses on the Agilent Tapestation with high sensitivity DNA screentape (D1000). Equal molar concentrations were multiplexed at a final concentration of 10 nM and sequenced using single-ended reads on an Illumina Hiseq4000 system. Due to limitations in the number of samples that could be combined in a single multiplex for sequencing, WT samples were independently multiplexed with *Ccnc* KO samples, and again with *Cdk8* KO samples, in order to eliminate any sequencing-run bias.

#### RNA-seq data analysis

FASTQ files were first checked with FASTQC (version 0.11.5, http://www.bioinformatics.babraham.ac.uk/projects/fastqc/ (45)) for quality control. The adaptor sequences were then removed using Trimmomatic (version 0.38 (46)), specifying the TruSeq3-SE.fa:2:30:10 adapters in the ILLUMINACLIP argument. The trimmed sequences were then aligned to the reference *Mus musculus* genome and annotation (build GRCm38.p6, NCBI:GCA_000001635.8) using HISAT2 (version 2.1.0 (47)) with default settings. Gene expression levels were then quantified from the aligned reads using featureCounts (version 2.0.1 (48)) with the options –ignoreDup and -s 2. Reference annotation GTF file was also obtained from genome build GRCm38.p6. Lastly, the R package DESeq2 (49) was used for exploratory data analysis and visualisation, as well as differential expression analyses. Reference genome sequence and annotation files were obtained from Ensembl (50).

#### Statistical analyses

Statistical tests were performed as indicated in the figure legends to determine statistical significance in replicate comparisons. A *P*-value < 0.05 was deemed statistically significant.

## RESULTS

### Identification and validation of factors influencing ATRi sensitivity in ATM-proficient and ATM-deficient cells

**Figure 1.**
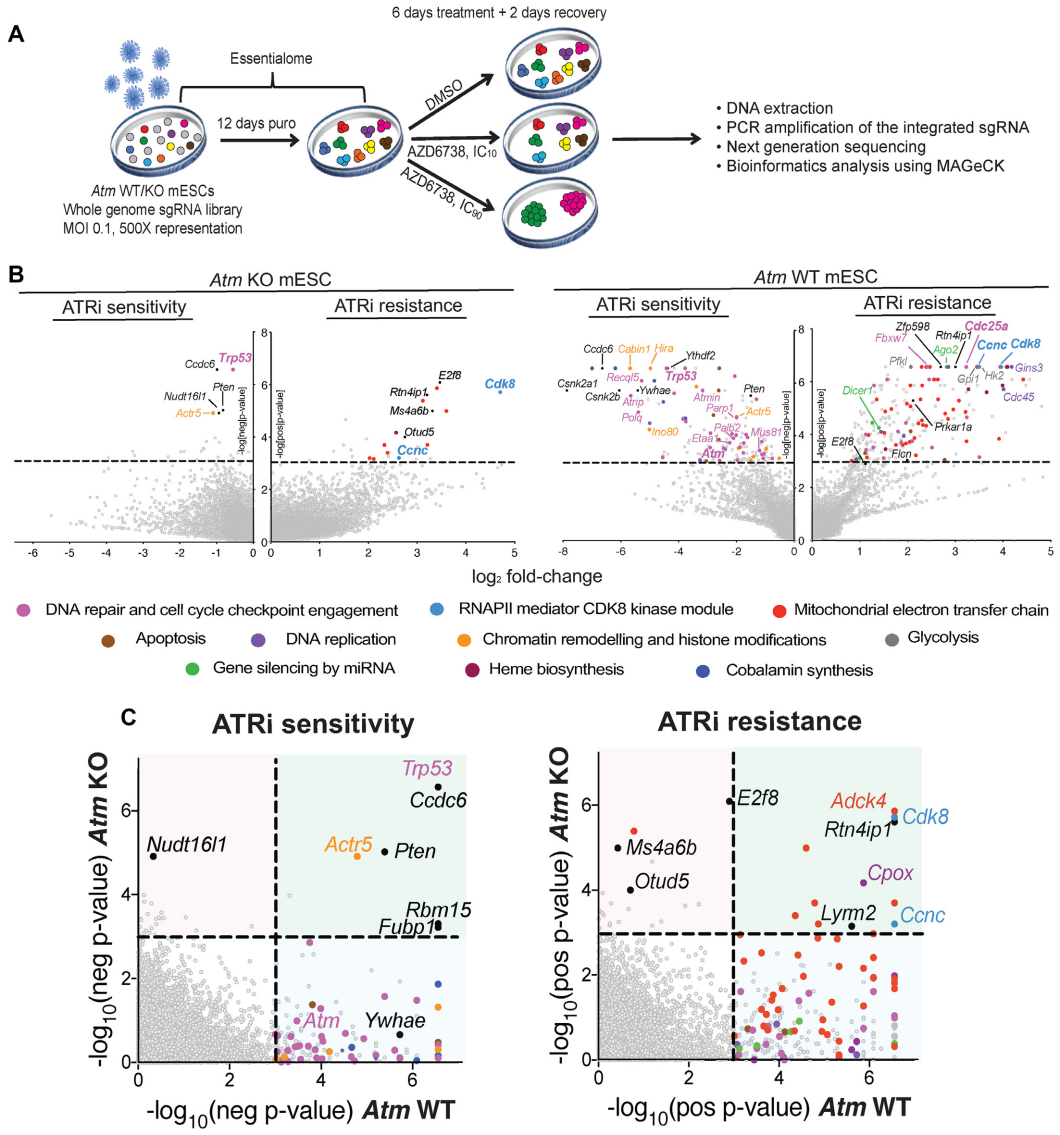
CRISPR–Cas9 screens using AZD6738 in *Atm* WT and KO mESCs. (**A**) Schematic of CRISPR–Cas9 screens performed in this study. (**B**) Drop-out and enrichment analyses following AZD6738 treatment in *Atm* WT and KO mESCs. Genes were statistically ranked using MAGeCK analysis software, and top hits with a *P*-value < 0.001 classified into related pathways and complexes. Dashed line = *P*-value of 0.001. Two MAGeCK analysis methods were performed for both IC_10_ and IC_90_ doses, and all analyses can be found in Supplementary Table S1 and Supplementary Figure S2A. The analyses that provided the greatest number of significant drop-out or enrichment hits (FDR < 0.1) for each genotype are presented. (**C**) Drop-out and enrichment analyses following AZD6738 treatment from Figure 1B, comparing MAGeCK *P*-values for each gene between *Atm* WT and *Atm* KO mESCs. A *P*-value of 0.001 is indicated by the dotted line. Genes in the upper right quadrant were hits in both cells lines, upper left quadrant were hits only in *Atm* KO cells, and those in the bottom right quadrant were hits only in *Atm* WT cells. Top hits in one or both cell lines are colour coded based on the same classification as Figure 1B.

To systematically interrogate drivers of ATRi efficacy in a clinically relevant genetic background, we performed whole genome CRISPR–Cas9 genetic screens in an isogenic pair of *Atm* WT and KO mESCs with a view to finding gene-products that, when lost, caused either hypersensitivity or resistance to the ATRi AZD6738 (36) (Figure 1A). We chose mESCs as our cell model in part because, compared to other cell models that we tested, ATM loss had minimal impact on cell proliferation rates (Supplementary Figure S1A), thereby facilitating the comparison of hits obtained between each genotype. Furthermore, the faster proliferation rates of mESCs ensured that cells underwent multiple rounds of replication and cell divisions in a 6-day treatment window. This was important because ATRi efficacy is likely to be influenced by S-phase drug exposure and subsequent mitotic entry (Supplementary Figure S1A). To enhance our prospects for uncovering both resistance and sensitivity factors, and to facilitate comparisons between both genetic backgrounds, we performed parallel screens at doses of AZD6738 that we had determined to equate to the IC_10_ and IC_90_ values for each cell line (as expected, *Atm* KO mESCs were significantly more sensitive to AZD6738 treatment than their WT counterparts; Supplementary Figure S1B). Following successful quality control (QC) of our screens (Supplementary Figure S1C–H), ensuing MAGeCK (51) bioinformatics analyses revealed a variety of factors and pathways predicted to contribute to ATRi hypersensitivity or resistance (Figure 1B, Supplementary Figure S2A, Supplementary Table S1). Furthermore, previously reported genes modulating sensitivity in *Atm* WT cells served as positive controls, indicating the reliability of our screens (*Cdc25a* for resistance (24); *Atm* and *Trp53* for sensitivity (14–16,52)). Notably, some gene-products whose inactivation was recently reported to hypersensitise cells to ATR inhibition (RNASEH2A, RNASEH2B, ERCC1, POLE3 and POLE4 (21–23)) behaved as essential in our screening conditions in mESCs, likely explaining our inability to detect these (Supplementary Figure S2B). To more easily compare hits obtained through our screens in *Atm* KO versus WT mESCs, we directly compared the *P*-values for each gene between both genotypes (Figure 1C) and generated a comprehensive list of genes predicted to enhance or suppress ATRi efficacy in *both* ATM-deficient and ATM-proficient cells (Supplementary Table S3).

**Figure 2.**
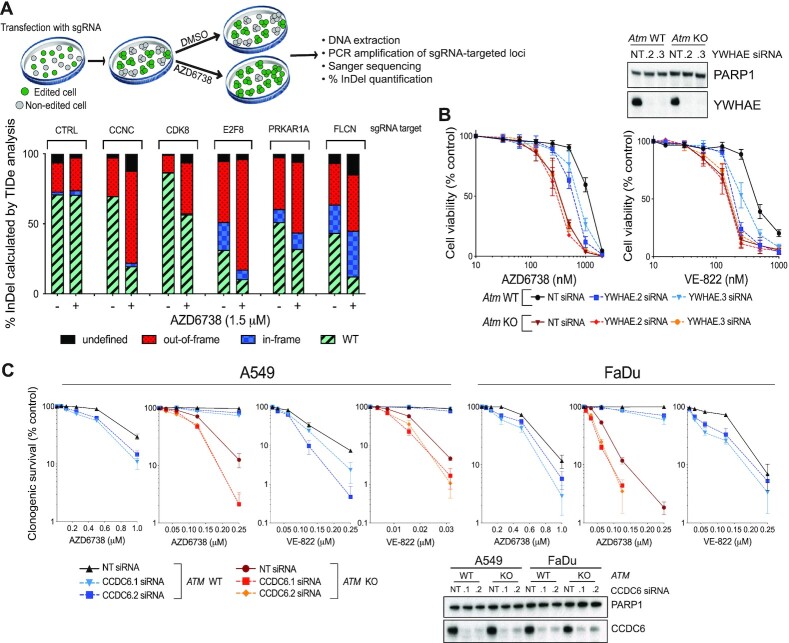
Validation of hits identified through our CRISPR–Cas9 screens. (**A**) Competition assays between CRISPR–Cas9 edited and non-edited cells in response to AZD6738 treatment. Cas9-expressing mESCs were transfected with synthetic sgRNAs against the target loci, resulting in a mixed pool of edited and non-edited cells after 7 days. Each pooled population was treated for 5 days with DMSO or 1.5 μM AZD6738, with a high dose strongly enriching for resistant cells. Genomic DNA was extracted and the sgRNA-targeted loci PCR amplified to allow editing efficiencies and % InDels (–50 bp to +50 bp) to be calculated using TIDe software (3.2.0) (44). The control sgRNA targeted *Lrrc29* which was not predicted to influence ATRi sensitivity in *Atm* WT mESCs according to our screening data. (**B**) Results of MTT cell proliferation assays in *Atm* WT and KO mESCs following siRNA-depletion of YWHAE, in response to two chemically distinct ATR inhibitors (AZD6738 and VE-822). Error bars = mean ± SEM (biological *n* = 3). (**C**) Clonogenic survivals of *ATM* WT and KO A549 and FaDu cells following siRNA-depletion of CCDC6, in response to two chemically distinct ATR inhibitors (AZD6738 and VE-822). Error bars = mean ± S.D (biological *n* = 3). FaDu *ATM* KO cells are inherently extremely sensitive to doses of VE-822 which effectively inhibit ATR kinase activity, therefore precluding analysis of further reductions in clonogenic survival upon CCDC6 depletion.

Two of the strongest resistance gene hits in the *Atm* KO mESCs were *Ccnc* and *Cdk8*, which encode two components of the same complex that regulates RNAPII-mediated transcription. Single guide-RNAs (sgRNAs) targeting both genes were also strongly enriched in the screen outputs from *Atm* WT cells, thereby highlighting the importance of this pathway in driving AZD6738 efficacy across multiple genetic backgrounds. Using CRISPR-based cell-growth competition assays (44,53), we provisionally validated both genes alongside various other resistance-gene hits from the *Atm* WT screens that commonly acquire missense mutations and/or reduced expression in cancer (*Prkar1a* (54–56)*, Flcn* (57,58)*, E2f8, Ccnc* (59) and *Cdk8* (60)) (Figure 2A). We therefore speculate that such mutations might represent secondary mutations in tumours that may appear unconnected to DNA replication and repair yet limit ATRi efficacy in the clinic.

Notably, we identified substantially more sensitiser and resistance hits in the *Atm* WT background than in the *Atm* KO mESCs (Figure 1C). Importantly, this was not due to an inability to detect changes in cell fitness in *Atm* KO cells upon gene editing, since genes encoding DNA-PK (*Prkdc*) and components of the Fanconi anaemia pathway (FA) had a greater impact on cell fitness in *Atm* KO versus *Atm* WT mESCs in accordance with existing literature (37,61,62) (Supplementary Figure S2C). Moreover, we validated the ATM-status dependency of ATRi sensitivity using small interfering RNAs (siRNAs) against *Ywhae* (14–3–3ϵ) (one of the strongest dropouts in WT cells only) and *Ccdc6* (one of the strongest dropouts in both *Atm* WT and KO cells) in various ATM-deficient models, using two independent ATR inhibitors (AZD6738 and VE-822; Figure 2B and C). These data also supported the relevance of our screening outputs in mESCs for understanding ATRi efficacy in human cancer cell models. Mechanistically, hits that we selectively detected in *Atm* WT mESCs could indicate proteins involved in ATM-regulated pathways that are therefore epistatic with ATM loss. Alternatively, finding fewer resistance hits in *Atm* KO compared to WT cells may highlight the existence of numerous pathways that are responsible for ATRi hypersensitivity in ATM-deficient cells. This in turn would suggest that patients with tumours harbouring ATM defects will be less likely to possess innate or readily acquire resistance to AZD6738 treatment through downregulation of, or loss-of-function mutations in, a single other gene.

Taken together, these data underscored successful systematic identification of factors and pathways promoting sensitivity and resistance to AZD6738, and highlighted how these vary depending on the cell's ATM status.

### Loss of Cyclin C or CDK8 provides ATRi resistance

**Figure 3.**
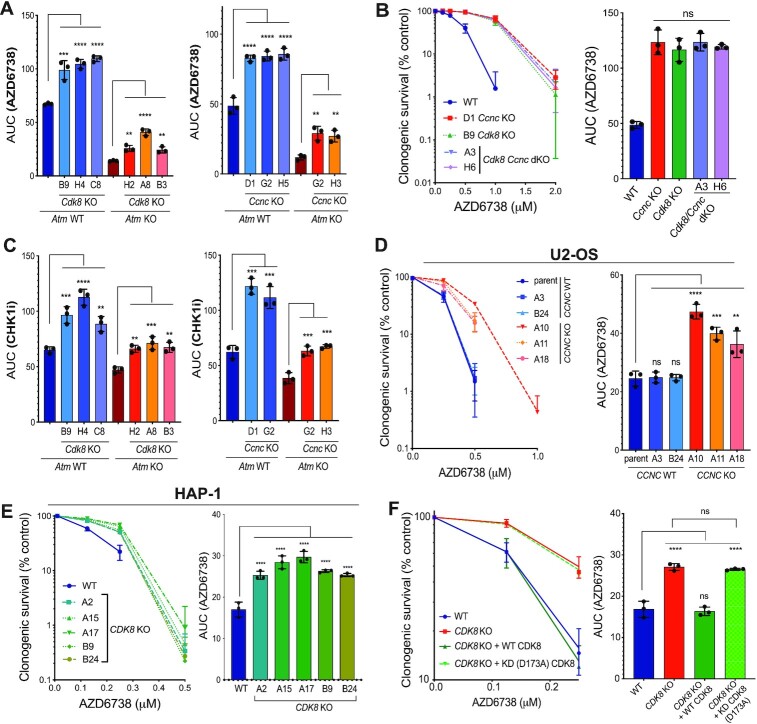
Loss of Cyclin C/CDK8 provides ATRi and CHK1i resistance. (**A**) Clonogenic survivals of *Ccnc*/*Cdk8* WT/KO *Atm* WT/KO mESCs treated with AZD6738, represented as AUCs (areas under the curve). Error bars = mean ± S.D (biological n = 3). Survival curves can be found in Supplementary Figure S3D. (**B**) Clonogenic survivals of WT and *Ccnc*/*Cdk8* single and dual KO mESCs treated with AZD6738. Data also represented as AUCs. Error bars = mean ± SD (biological *n* = 3). (**C**) Clonogenic survivals of *Ccnc*/*Cdk8* WT/KO *Atm* WT/KO mESCs treated with the CHK1 inhibitor LY2603618, represented as AUCs. Error bars = mean ± SD (biological *n* = 3). Survival curves can be found in Supplementary Figure S3F. (**D**) Clonogenic survivals of WT and *CCNC* KO U2-OS cells in response to AZD6738 treatment. A3/B24 clones were confirmed as WT by immunoblot analyses and genotyping after single-cell expansions of sgRNA-transfected cells. Data also represented as AUCs. Error bars = mean ± SD (biological *n* = 3). (**E**) Clonogenic survivals of WT and *CDK8* KO HAP-1 cells in response to AZD6738 treatment. Data also represented as AUCs. Error bars = mean ± SD (biological *n* = 3). (**F**) Clonogenic survivals of WT and *CDK8* KO HAP-1 cells, complemented with WT or kinase dead (D173A) CDK8-GFP, treated with AZD6738. Data also represented as AUCs. Error bars = mean ± SD (biological *n* = 3). Additional clones are shown in Supplementary Figure S5A and B. All statistical analyses were performed using a one-way ANOVA test with multiple comparisons. *P*-values < 0.05 (*), 0.01 (**), 0.001 (***) and 0.0001 (****) were deemed statistically significant.

For ensuing mechanistic follow up studies, we focussed on two of the strongest resistance gene hits that were common genetic modulators of ATRi efficacy in both *Atm* KO and WT backgrounds: the genes encoding Cyclin C (*Ccnc)* and cyclin-dependent kinase 8 (*Cdk8)*, which comprise part of the CDK8 kinase module (CKM) for the RNAPII mediator complex (Figure 1C). *De novo* CRISPR-mediated gene KO of either *Ccnc* or *Cdk8* in both *Atm* WT and KO mESCs (Supplementary Figure S3A–C) produced resistance to AZD6738 treatment, thereby further validating our screening results (Figure 3A, Supplementary Figure S3D). Importantly, concomitant knockout of *Ccnc* and *Cdk8* yielded no additive resistance, suggesting that they function in a common pathway (Figure 3B, Supplementary Figure S3E). Supporting this, we noticed that *Cdk8* KO cells also had reduced Cyclin C levels (Supplementary Figure S3A, B), consistent with a previous report showing that CDK8 is required for Cyclin C stability, but not vice-versa (63).

Furthermore, we found that loss of Cyclin C or CDK8 conferred resistance to a clinical ATRi, VE-822, that is chemically distinct from AZD6738, as well as towards the CHK1i (LY2603618) or WEE1i (AZD1775), but not towards various DNA-damaging agents that we tested: ionising radiation (IR) which causes various forms of DNA damage, the strong apoptosis inducers etoposide and carboplatin, as well as hydroxyurea (HU) or aphidicolin which induce DNA replication stress by impairing DNA polymerase processivity (Figure 3C, Supplementary Figure S3F–J). This indicated that the resistance mechanism afforded by loss of Cyclin C or CDK8 appeared specific to inhibition of the RSR but did not act more generally in response to other sources of replication stress or DNA damage. Notably, Cyclin C and CDK8 have been reported to upregulate the transcription of a subset of p53-dependent genes (64). However, rather than causing resistance, p53 loss caused sensitivity to AZD6738, consistent with previous reports (15,16,52). Given that CKM positively regulates the p53 downstream responses, this is unlikely to underpin the mechanism of ATRi resistance driven by CKM loss. To further support this notion, we deleted *CCNC* or *CDK8* in human U2-OS and HAP-1 cells respectively (Supplementary Figure S3K-L)—two cell lines that display dysfunctional p53 signalling (Supplementary Figure S3M). Loss of Cyclin C or CDK8 in these cell lines also caused AZD6738 resistance (Figure 3D and E), thereby supporting a p53-independent mechanism and corroborating our findings in human cancer cell lines. Importantly, unperturbed Cyclin C- or CDK8-deficient cells showed no discernible difference in cell cycle profiles or growth kinetics compared to their WT counterparts in all three cellular backgrounds (Supplementary Figure S4A–C).

To assess whether CDK8 kinase activity is required for AZD6738 treatment response, we complemented *CDK8* KO HAP-1 cells with either a WT CDK8-GFP construct, or one bearing a D173A mutation that inactivates CDK8 catalytic activity (65). We observed that the D173A mutant provided ATRi resistance comparable to complete CDK8 loss, suggesting that CDK8 kinase activity is critical for its function in regulating ATRi sensitivity (Figure 3F, Supplementary Figure S5A-B). Accordingly, we observed that CDK8 kinase activity was abrogated to a similar extent in both Cyclin C and CDK8 deficient cells, as measured by probing for phosphorylation of CDK8 targets STAT1 (pS727) and STAT5 (pS726) (66,67) (Supplementary Figure S5C; as expected, CDK8 activity was also restored by complementing cells with WT, but not D173A mutant, CDK8).

### Cyclin C/CDK8 loss modulates ATRi sensitivity independently of CDC25A and G2/M checkpoint function

**Figure 4.**
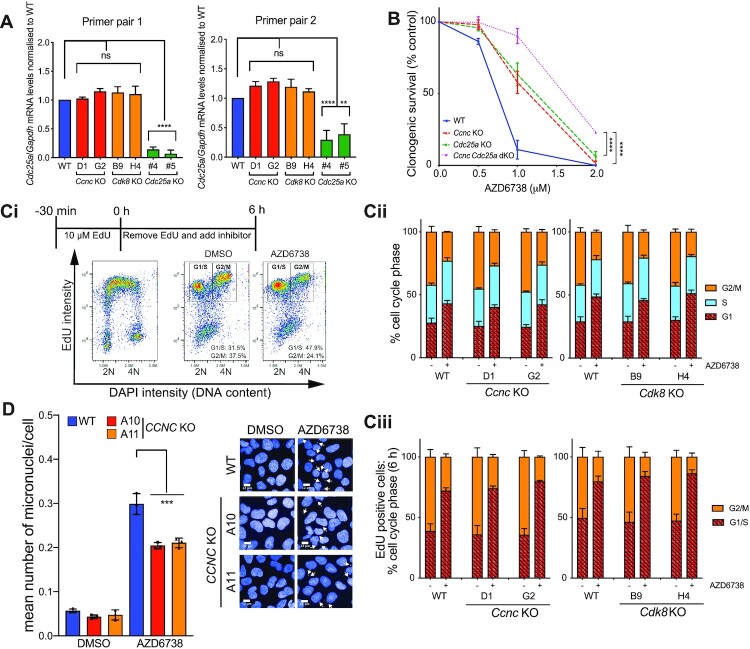
Loss of Cyclin C/CDK8 promotes ATRi resistance independent of CDC25A or G2/M checkpoint regulation. (**A**) *Cdc25a* mRNA transcript levels in WT and *Ccnc/Cdk8* KO mESCs as assessed by RT-qPCR using two independent primer pairs. Transcript levels are normalised to the house-keeping gene *Gapdh*. *Cdc25a* KO mESCs (24) were used as a control. Error bars = mean ± SEM (biological *n* = 4 WT, *Cdk8* KO, *Ccnc* KO; *n* = 2 *Cdc25a* KO). (**B**) Clonogenic survivals of isogenic WT and *de novo* generated *Ccnc*/*Cdc25a* single and dual KO mESCs treated with AZD6738. Error bars = mean ± SD (biological *n* = 3). (**C**) Actively replicating S-phase *Ccnc* and Cdk8 WT and KO mESCs were pulse-labelled with 10 μM EdU for 30 min prior to release into media containing DMSO or 900 nM AZD6738 for 6 h. Error bars = mean ± SEM (biological *n* = 3 *Ccnc* KO, *n* = 4 *Cdk8* KO). (i) Schematic of experimental design and gating strategy using FACS profiles in WT mESCs. (ii) Cell cycle distributions based on DAPI histograms. iii) Distribution of EdU positive cells between G1/S and G2/M 6 h after removal of EdU. DNA content 2N = G1, 4N = G2/M. EdU positive cells were actively replicating at the time of EdU labelling. (**D**) Mean number of micronuclei/cell after 24 h treatment with DMSO or 1.5 μM AZD6738 in U2-OS *CCNC* WT and KO cells. Error bars = mean ± SD (biological *n* = 3). Arrows indicate micronuclei in the representative images. Representative images were taken at 40× magnification, scale bars = 12 μm. All statistical analyses were performed using a one-way ANOVA test with multiple comparisons, except in Figure 3B where a two-way ANOVA test was used. *P*-values < 0.05 (*), 0.01 (**), 0.001 (***) and 0.0001 (****) were deemed statistically significant.

CDC25A loss has been reported to provide ATRi resistance by opposing the G2/M checkpoint override and premature mitotic entry caused by ATRi treatment (24). We therefore sought to establish whether Cyclin C and CDK8 were epistatic with CDC25A regarding ATRi sensitivity. Firstly, we assessed *Cdc25a* transcript levels in *Cdk8* KO and *Ccnc* KO mESCs, and found no change compared with the WT cells (Figure 4A). Furthermore, *de novo* CRISPR-mediated knockout of *Cdc25a* in *Ccnc* WT or *Ccnc* KO backgrounds demonstrated a clear additive effect of inactivating each gene on AZD6738 resistance, consistent with Cyclin C and CDC25A acting in different pathways to promote AZD6738 resistance (Figure 4B). To test whether loss of Cyclin C/CDK8 suppressed AZD6738-induced G2/M checkpoint abrogation via an alternate pathway, we performed pulse-chase experiments with the nucleotide analogue EdU (Figure 4Ci). As expected, incubation with AZD6738 caused faster progression of cells though mitosis and into G1 phase, as indicated by the boxes in Figure 4Ci, and quantified in Figure 4Cii-iii). Importantly, progression rates from G2 to G1 were not discernibly different between WT, *Ccnc* KO and *Cdk8* KO mESCs, when assessed in either the presence or absence of AZD6738 (Figure 4Cii–iii). However, despite the G2/M checkpoint being equally defective, we noted that micronuclei levels following 24 h treatment with AZD6738 were significantly reduced in *CCNC* KO U2-OS cells as compared to WT controls (Figure 4D). These data therefore suggested that when ATR is inhibited, Cyclin C/CDK8 promote increased DNA damage that persists until mitosis, wherein it leads to increased micronuclei formation.

### Cyclin C loss reduces basal and ATRi/CHK1i-induced DNA:RNA hybrids

CDK8 kinase activity is primarily associated with phosphorylating the C-terminal domain (CTD) of RNAPII and various transcription factors (68), therefore pointing towards transcriptional control as the mechanism by which Cyclin C/CDK8 loss promotes ATRi resistance. Previous studies on CKM in mammalian cells have established that its depletion leads to transcriptome changes but not a genome-wide reduction in RNAPII activity (69–71). In line with this, we observed no consistent reduction in mRNA synthesis or abundance of elongating RNAPII in our Cyclin C/CDK8-deficient cells as compared to controls (Supplementary Figure S6A-F). We therefore probed for specific changes in the transcriptome by performing RNA-seq in WT, *Cdk8* KO and *Ccnc* KO mESCs following DMSO or AZD6738 treatment. While quality control metrics and biological controls supported the reliability of our data (Supplementary Figure S6G–J), we did not identify any transcripts that were significantly differentially regulated upon AZD6738 treatment in both *Ccnc* and *Cdk8* KO, but not WT, cells (Supplementary Table S4). Similarly, Gene Ontology analyses of proteins whose transcripts were upregulated or downregulated in a treatment-independent manner in both *Cdk8* and *Ccnc* KO clones compared to WT mESCs did not provide a clear explanation why the *Ccnc*/*Cdk8* KO cells are ATRi resistant (Supplementary Table S4).

**Figure 5.**
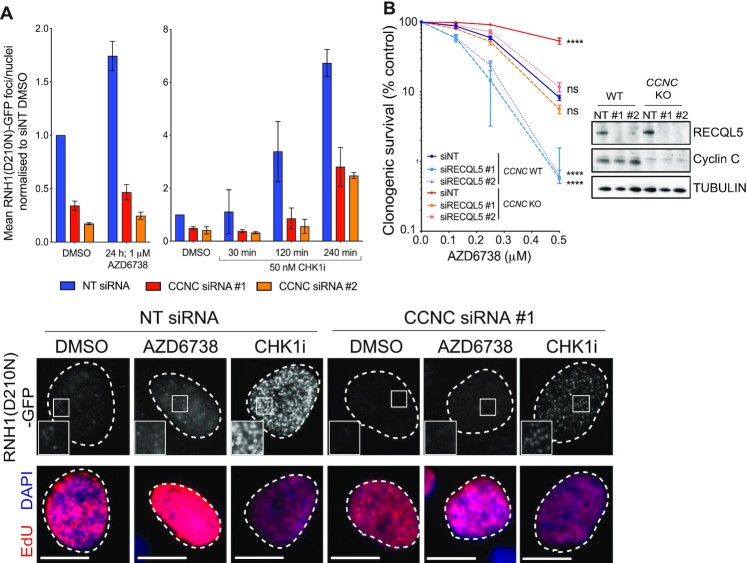
Cyclin C loss supresses DNA:RNA hybrid formation in response to ATRi and CHK1i. (**A**) Mean number of RNH1(D210N)-GFP foci/nuclei normalised to untreated NT siRNA levels in response to AZD6738 or CHK1i (LY2606368) treatment, following siRNA-depletion of Cyclin C. Error bars = mean ± SD (biological *n* = 2). Representative images are of RNH1(D210N)-GFP foci in EdU positive cells, following treatment with DMSO, AZD6738 for 24 h, or CHK1i for 4 h. Scale bars = 12 μm. A full field of view is provided in Supplementary Figure S7C which also highlights the heterogeneity of foci induction in Cyclin C-depleted cells treated with CHK1i. SiRNAs targeting CDK8 were also tested but resulted in the accumulation of cells in G1 which may bias the experimental outcome (Supplementary Figure S7A and B). (**B**) Clonogenic survivals of RECQL5-depleted WT and *CCNC* KO U2-OS cells treated with AZD6738. Error bars = mean ± SD (biological *n* = 3). Statistical analyses were performed using a two-way ANOVA test by comparison to *CCN*C WT cells transfected with the control (NT) siRNA. *P*-values < 0.05 (*), 0.01 (**), 0.001 (***) and 0.0001 (****) were deemed statistically significant.

Instead, we speculated that loss of Cyclin C/CDK8 may reduce collisions between the transcription and replication machineries upon ATR inhibition, whereby transcription-replication conflicts are associated with DNA:RNA hybrid formation and genome instability, primarily through causing replication fork collapse (9,72). We therefore used a reporter system employing catalytically dead RNase H1 (RNH1(D210N) (39))—which binds to but does not process DNA:RNA hybrids - to quantify DNA:RNA hybrid levels in response to ATR or CHK1 inhibition following depletion of Cyclin C. Since CDK8 depletion caused significant accumulation of U2-OS cells in G1 and also reduced Cyclin C protein levels (63) (Supplementary Figure S3A-B and Supplementary Figure S7A and B), we focused largely on Cyclin C loss for further studies. Strikingly, this revealed that both basal and ATRi/CHK1i-induced DNA:RNA hybrid levels were markedly reduced in cells depleted of Cyclin C (Figure 5A; as shown in Supplementary Figure S7C, DNA:RNA hybrids formed in each case in S-phase cells). In light of these data, we hypothesised that Cyclin C loss alleviated the rate of transcription-replication encounters that are enhanced upon ATRi or CHK1i treatment, as opposed to promoting DNA:RNA hybrid resolution. Supporting this, RECQL5, which is reported to limit and resolve transcription-replication conflicts (73,74), was identified as an ATRi sensitiser in our CRISPR–Cas9 screens (Figure 1B). Depletion of RECQL5 enhanced sensitivity to AZD6738 and rescued the ATRi resistance observed upon Cyclin C loss, with *CCNC*-knockout RECQL5-depleted cells showing equivalent sensitivity to WT cells transfected with a control siRNA (Figure 5B). Taken together, these results showed that Cyclin C loss counteracts DNA:RNA hybrid formation caused by ATR and CHK1 inhibition in S-phase, suggesting that counteracting or reducing transcription-replication conflicts improved cell survival.

### Loss of Cyclin C suppresses replication stress in response to ATR or CHK1 inhibition

**Figure 6.**
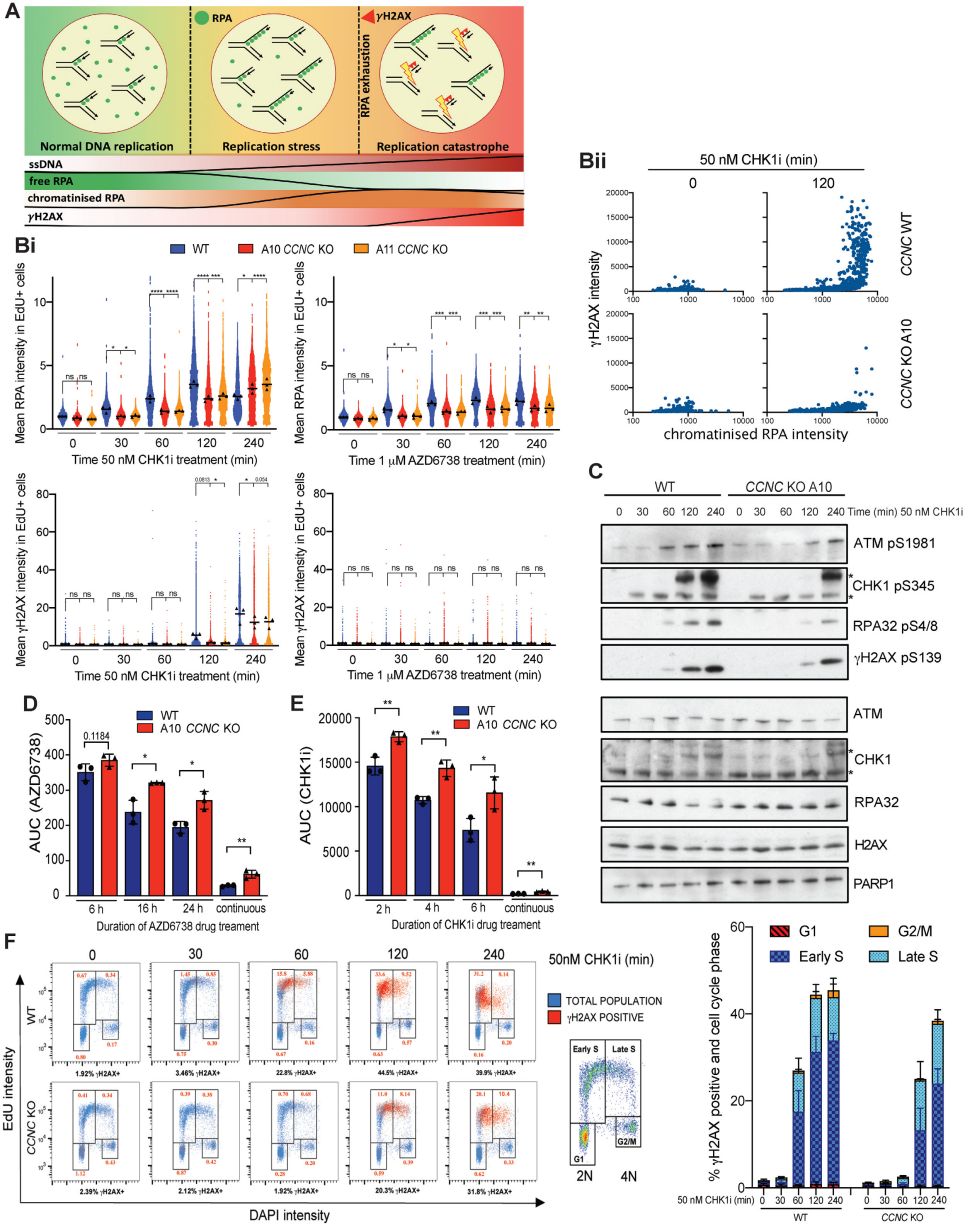
Cyclin C loss supresses replication stress in response to ATRi and CHK1i. (**A**) Schematic of replication stress and replication catastrophe, which can be monitored by increased RPA32 chromatinisation (replication stress), followed by detection of γH2AX in RPA32 hyper-positive cells at later time points (replication catastrophe). (**B**) Chromatinised RPA32 and γH2AX intensities in EdU positive nuclei measured by immunofluorescence in *CCNC* WT and KO U2-OS cells. S-phase cells were labelled with 10 μM EdU for 30 min prior to pre-extraction and fixation. Representative images can be found in Supplementary Figure S8A. (i) Mean intensities normalised to non-treated WT cells, following 0–240 min treatment with 50 nM CHK1i (LY2603638) or 1 μM AZD6738 (biological *n* = 3). Mean intensities for each replicate are displayed as black triangles and were used for the overall mean calculations and statistical analyses. Mean intensities for each individual cell were normalised to the mean intensity of non-treated WT cells in each replicate, and all three replicates overlaid in blue (*CCNC* WT), red (*CCNC* KO A10) or orange (*CCNC* KO A11) for visualisation of single-cell data. These data are also normalized to the non-treated conditions of each individual cell line (Supplementary Figure S8B) to highlight that the suppression of replication stress upon ATRi/CHK1i in *CCNC* KO cells is independent of any differences in basal intensities. Statistical analyses were performed using one-way ANOVA analyses with multiple comparisons. *P*-values < 0.05 (*), 0.01 (**), 0.001 (***) and 0.0001 (****) were deemed statistically significant. ii) Representative example of replication catastrophe occurring in *CCNC* WT, but not KO, cells following 120 min CHK1i treatment. Mean chromatinised RPA32 and γH2AX intensities for each EdU-positive cell are presented as a scatter plot. (**C**) Immunoblots for markers of DSB formation, indicative of replication catastrophe. *CCNC* WT and KO U2-OS cells were treated for 0–240 min with 50 nM CHK1i (LY2603638) prior to lysis. *Both bands are modified forms of CHK1 as indicated by the shift in total protein. (**D**) Clonogenic survivals of *CCNC* WT and KO U2-OS cells treated with AZD6738 for 6, 16 or 24 h prior to drug wash-out, represented as AUCs. Error bars = mean ± SD (biological *n* = 3). Survival curves can be found in Supplementary Figure S8G. Statistical analyses were performed using a two-tailed unpaired Student's *t*-test. *P*-values < 0.05 (*), 0.01 (**), 0.001 (***) and 0.0001 (****) were deemed statistically significant. (**E**) Clonogenic survivals of *CCNC* WT and KO U2-OS cells treated with CHK1i LY2603638 for 2, 4 or 6 h prior to wash-out, represented as AUCs. Error bars = mean ± SD (biological *n* = 3). Survival curves can be found in Supplementary Figure S8H. Statistical analyses were performed using a two-tailed unpaired Student's *t*-test. *P*-values < 0.05 (*), 0.01 (**), 0.001 (***) and 0.0001 (****) were deemed statistically significant. (**F**) Representative FACS plots gated for cell-cycle phase using EdU versus DAPI intensity (blue), overlaid with γH2AX positive cells (red) after 0–240 min treatment of *CCNC* WT and KO U2-OS cells with 50 nM CHK1i (LY2603638). DNA content 2N = G1, 4N = G2/M. S-phase cells are EdU positive following 30 min treatment with 10 μM EdU, and are gated into early (2N–3N) or late (3N–4N) S-phase. Numbers in red indicate the % of the total population that are γH2AX positive and in the associated cell-cycle phase. A representative gating strategy for γH2AX positive cells is shown in Supplementary Figure S8I. Quantification of % cells that are γH2AX positive and in the associated cell-cycle phase in *CCNC* WT and KO cells following CHK1i treatment is also provided. Error bars = mean ± SD (biological *n* = 3).

Both transcription-replication conflicts and DNA:RNA hybrids have been established as sources of genome instability, largely through promoting replication fork stalling and collapse into single-ended DSBs (seDBSs) (9,10). In the absence of replication fork protection, replication stress can also cause RPA exhaustion, leading to nuclease processing of exposed ssDNA, global replication fork breakage and ATM activation (11). Importantly, ATR and CHK1 play pivotal roles in counteracting this phenomenon, known as replication catastrophe, by limiting global origin firing and promoting nucleotide synthesis. We therefore assessed whether Cyclin C loss decreased replication stress and ultimately prevented replication catastrophe in the presence of ATRi or CHK1i. To assess this, we monitored RPA32 chromatinisation and histone H2AX Ser-139 phosphorylation (γH2AX) over time in *CCNC* WT and KO U2-OS cells treated with ATRi or CHK1i. RPA32 hyper-positive cells were indicative of replication stress, while pan-nuclear RPA32 and γH2AX dual-positive cells detected at later time points suggested replication catastrophe (11) (Figure 6A). As expected, we found that RPA32 and γH2AX accumulation occurred primarily in EdU positive cells, confirming that active DNA replication in S-phase is required for DNA damage generation. We observed that Cyclin C loss reduced replication stress (RPA chromatinisation) induced by either ATR or CHK1 inhibition (Figure 6Bi; Supplementary Figure S8A and B). In *CCNC* KO cells, ATRi-induced replication stress remained below that observed in WT cells for the full 24 h treatment window tested (Supplementary Figure S8C), with this reduction presumably sufficient to limit under-replicated DNA from entering mitosis as indicated by the reduced micronuclei formation in *CCNC* KO cells at 24 h (Figure 4D). On the other hand, CHK1i-induced replication stress resulted in replication catastrophe, consistent with the more potent induction of DNA:RNA hybrid formation following treatment with CHK1i compared with ATRi treatment. In agreement with the reduction in DNA:RNA hybrids observed in Cyclin C-depleted cells, we observed a delayed induction of replication catastrophe in *CCNC* KO compared to WT cells upon CHK1 inhibition (Figure 6Bi-ii). In line with this, in *CCNC* KO cells we observed delayed kinetics of RPA32 phosphorylation on Ser-4/8, which has been associated with replication fork collapse and seDSB (single-ended DSB) formation (11,75), as well as delayed ATM activation detected by its auto-phosphorylation on Ser-1981 (Figure 6C). This occurred in a dose-dependent manner, with *CCNC* KO cells experiencing a delayed onset of DNA damage by several hours at lower CHK1i doses (Supplementary Figure S8D and E). A similar delay in γH2AX formation and Ser-4/8 RPA32 phosphorylation was observed in *Ccnc* and *Cdk8* KO mESCs as compared to WT control cells (Supplementary Figure S8F). Importantly, given that Cyclin C loss only delayed the onset of CHK1i-induced replication catastrophe, we assessed whether this was sufficient to increase cell survival. Indeed, short-term treatment with either ATRi or CHK1i still induced cell death in *CCNC* WT cells, whereas *CCNC* KO cells retained the resistance phenotype observed with continuous ATRi or CHK1i treatment (Figure 6D and E, Supplementary Figure S8G and H).

To assess at which stage in S-phase DNA damage was occurring in our studies, we probed for γH2AX following CHK1 inhibition using flow cytometry and gated the cells as early or late S-phase based on EdU and DAPI intensity. This revealed that CHK1i-treated *CCNC* WT cells accumulated with DNA damage in early S-phase, whereas in *CCNC* KO cells γH2AX formed only at later time points and in both early and late S-phase (Figure 6F, Supplementary Figure S8I). In *CCNC* WT cells, this was associated with a dramatic reduction in EdU incorporation by 240 minutes, indicating that any cells capable of entering mitosis would do so with an under-replicated genome. Conversely, despite accumulating DNA damage (γH2AX) by 240 min, *CCNC* KO cells appeared more likely to enter mitosis with a near-fully replicated genome, therefore supporting a model in which a delay in replication catastrophe is sufficient to promote cell survival. Together, these data suggested that Cyclin C loss in S-phase alleviates replication stress induced by ATR or CHK1 inhibition, and that this enhances cell survival.

### Cyclin C loss alleviates transcription-associated replication stress

**Figure 7.**
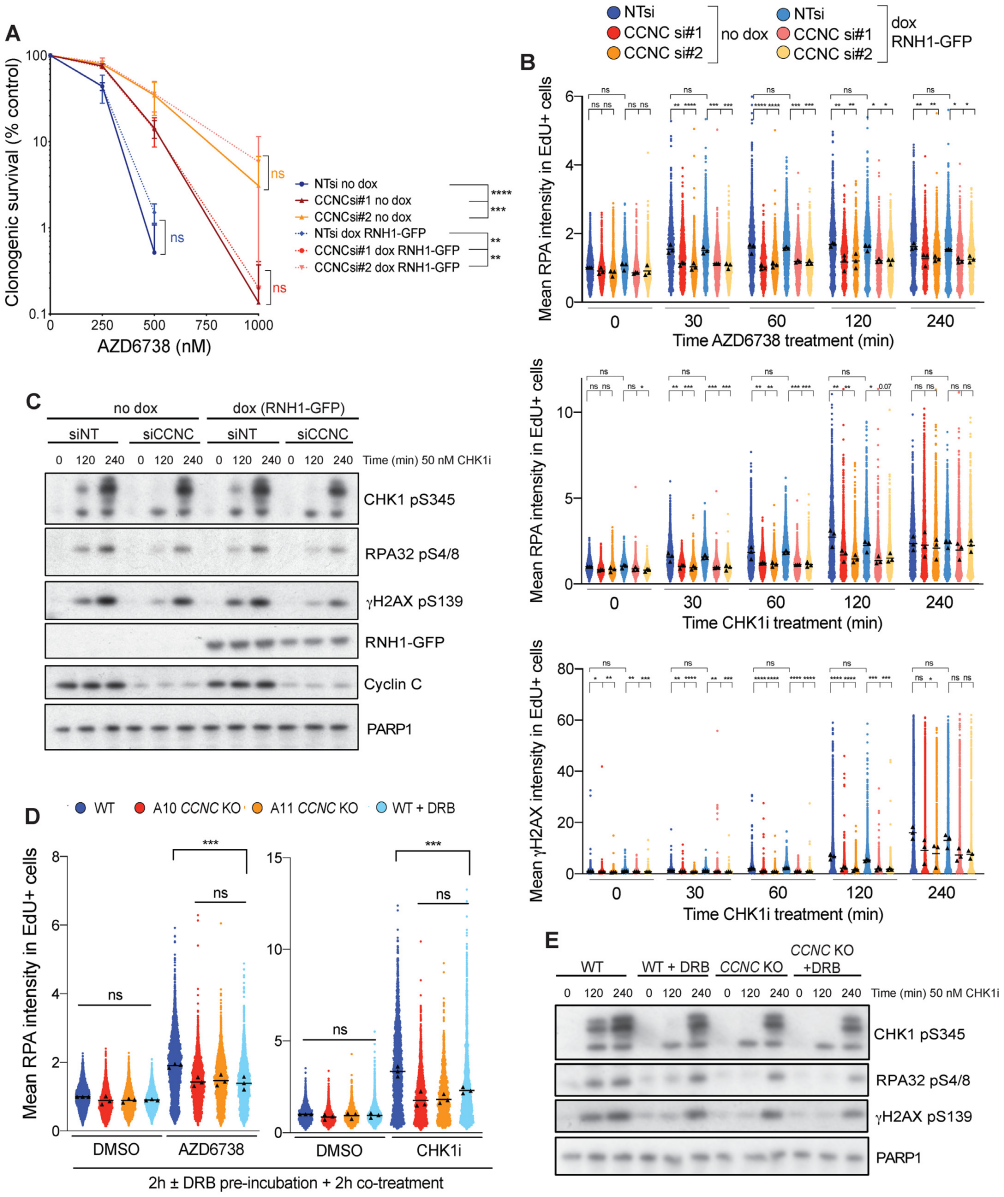
Cyclin C-associated replication stress is transcription-dependent. (**A**) Clonogenic survivals of U2-OS cells ± Cyclin C depletion ± doxycycline induction of RNH1-GFP, treated with AZD6738. Error bars = mean ± SD (biological *n* = 3). Statistical analyses were performed using two-way ANOVA analyses. *P*-values < 0.05 (*), 0.01 (**), 0.001 (***) and 0.0001 (****) were deemed statistically significant. (**B**) Mean chromatinised RPA32 and γH2AX intensities in EdU positive nuclei measured by immunofluorescence in WT or Cyclin C-depleted U2-OS cells, ± doxycycline-induced RNH1-GFP expression, treated for 0–240 min with 50 nM CHK1i (LY2603638) or 1 μM AZD6738. S-phase cells were labelled with 10 μM EdU for 30 min prior to pre-extraction and fixation. Mean intensities for each replicate are displayed as black triangles and were used for the overall mean calculations and statistical analyses. Mean intensities for each individual cell were normalised to the mean intensity of non-targeted siNT cells in each replicate, and all three replicates overlaid for visualisation of single-cell data. Statistical analyses were performed using one-way ANOVA analyses with multiple comparisons. *P*-values < 0.05 (*), 0.01 (**), 0.001 (***) and 0.0001 (****) were deemed statistically significant. (**C**) Immunoblots for markers of DSB formation, indicative of replication catastrophe. U2-OS cells were siRNA-depleted of Cyclin C, or transfected with the control NT siRNA, and RNH1-GFP expression induced by the addition of doxycycline 24 h prior to treatment with 50 nM CHK1i (LY2603638) for 0, 2 or 4 h. CCNC siRNA #1 was used here, and the results obtained using siRNA #2 are provided in Supplementary Figure S9B. (**D**) Mean chromatinised RPA32 intensities in EdU positive nuclei measured by immunofluorescence in *CCNC* WT and KO U2-OS cells, normalised to non-treated WT cells (biological *n* = 3). Cells were pre-incubated with DMSO or 100 μM DRB for 2 h prior to 2 h co-treatment with DMSO, 1 μM AZD6738 or 50 nM CHK1i (LY2603638). S-phase cells were labeled with 10 μM EdU for 30 min prior to pre-extraction and fixation. Mean intensities for each replicate are displayed as black triangles and were used for the overall mean calculations and statistical analyses. Mean intensities for each individual cell were normalised to the mean intensity of non-treated WT cells in each replicate, and all three replicates overlaid for visualisation of single-cell data. Statistical analyses were performed using one-way ANOVA analyses with multiple comparisons. *P*-values < 0.05 (*), 0.01 (**), 0.001 (***) and 0.0001 (****) were deemed statistically significant. (**E**) Immunoblots for markers of DSB formation, indicative of replication catastrophe. *CCNC* WT and KO U2-OS cells were pre-incubated with 100 μM DRB for 2 h prior to co-treatment with 50 nM CHK1i (LY2603638) for the time-periods indicated. Control cells were treated with DMSO in parallel with the 4 h CHK1i incubation.

Although DNA:RNA hybrid formation and transcription-replication conflicts are often linked, they are not a single event or interchangeably defined, with both being able to promote genome instability through replication fork collapse. Furthermore, DNA:RNA hybrids and transcription-replication conflicts can be both caused by, or cause, the other, and various sources of DNA:RNA hybrids have been identified (76). We therefore deemed it important to distinguish whether the Cyclin C associated replication stress arising from ATRi or CHK1i treatment was caused by collisions between transcription and replication machineries, the DNA:RNA hybrids, or both. To investigate whether the DNA:RNA hybrids were themselves the cause, we assessed the impact on ATRi/CHK1i-induced replication stress of overexpressing RNase H1 which degrades DNA:RNA hybrids (Supplementary Figure S9A). Through these experiments, we observed no significant impact of RNase H1 overexpression on cell survival, replication stress or replication catastrophe following CHK1i or ATRi treatment in the presence or absence of Cyclin C (Figure 7A–C, Supplementary Figure S9B). These data therefore indicated that the DNA:RNA hybrids themselves were likely not directly responsible for ATRi or CHK1i-induced replication stress and cell death, but instead suggested that the upstream physical collisions between transcription and replication machineries were promoting genome instability. Furthermore, Cyclin C depletion similarly rescued the impact of ATRi or CHK1i treatment in both RNase H1 un-induced and RNase H1 overexpressing cells, thereby supporting the idea that Cyclin C loss acts upstream to limit DNA:RNA hybrid formation rather than acting at the level of their resolution.

Finally, to assess a role for transcription-replication collisions in Cyclin C-associated replication stress, we pre-incubated cells with the transcription inhibitor DRB (7,77–79) at a dose and time-points that reduced transcription-replication collisions as indicated by a proximity ligation assay (PLA) between RNAPII and the DNA replication component PCNA, yet had minimal or no impact on the cell cycle (Supplementary Figure S9C–E). Accordingly, we found that DRB pre-treatment reduced ATRi- and CHK1i-induced RPA chromatinisation in *CCNC* WT cells to levels similar to those observed in *CCNC* KO cells (Figure 7D, Supplementary Figure S9F). We also observed a similar delay in the kinetics of γH2AX, CHK1 pSer-345 and RPA pSer-4/8 following CHK1i treatment in DRB pre-treated *CCNC* WT cells comparable with *CCNC* KO cells (Figure 7E). Notably, Cyclin C loss or DRB pre-treatment did not impact on IR-induced DNA damage as indicated by similar levels of γH2AX, CHK1 pSer-345 and ATM pSer-1981 (Supplementary Figure S9G). This is consistent with our extended cell survival data (Supplementary Figure S3J) and underlines a specific impact of Cyclin C on replication stress-induced DNA damage. Overall, these data supported a major role for transcription-associated replication stress in driving ATR and CHK1 inhibitor efficacy and highlight Cyclin C and CDK8 as key factors capable of modulating this response.

## DISCUSSION

ATR inhibitors are in clinical trials for the treatment of cancers (12), with patient selection hypotheses including ATM deficiency (13–16) and high replication stress (17–20). To identify mechanisms of ATRi efficacy in cells with clinically relevant mutations, we performed the first whole-genome ATRi CRISPR–Cas9 screens in paired isogenic ATM-proficient and ATM-deficient cells. Through this approach, we identified known as well as novel factors which, when lost, provided either sensitivity or resistance to ATRi in one or both genetic backgrounds. Of these hits, several modulated ATRi sensitivity independent of ATM expression, including *Trp53* and *Ccdc6* (sensitivity) and *Ccnc* and *Cdk8* (resistance). These genes highlight mechanisms of ATRi efficacy that may drive cell death across multiple tumour types. Notably, we identified substantially fewer gene hits in *Atm* KO compared to WT cells, which may reflect the numerous interplays between ATR and ATM to regulate DNA repair, DNA replication and cell cycle checkpoint engagement. It is therefore plausible that loss of a single protein is in most instances insufficient to promote ATRi resistance in ATM-deficient cells, as cell death can still be driven through deregulation of another critical pathway. By highlighting that it may be difficult to generate resistance phenotypes in this setting, our findings therefore support the targeted use of ATRi against tumours with impaired ATM function, and propose the attraction of inhibiting ATR rather than other DDR factors such as PARP in ATM-deficient tumours, where resistance can be imparted by single inactivation of various genes due to one primary mode of cell death (38). Alternatively, certain hits arising in our screens that increased sensitivity in *Atm* WT but not *Atm* KO cells may encode proteins that act in the same pathway as ATM to modulate ATRi efficacy, and therefore exhibit an epistatic relationship with ATM loss. Such components represent candidates for identifying ATM-mediated back-up pathways that can compensate in the absence of functional ATR. We note however that, in some cases, an increased impact on cell fitness in *Atm* KO mESCs may have affected our ability to detect drug-dependent changes in the sgRNA abundances of certain genes in *Atm* KO cells. We therefore encourage the cross-referencing of ATM-dependant hits with our essential-gene screen outcomes when prioritising candidates for further study. Nevertheless, through validating several such factors and their ATM-status dependency, we believe that our data provide a resource to inform future studies into interplays between the ATR and ATM kinases, as well as the clinical use of ATR inhibitors.

Our studies have shown that loss of Cyclin C or CDK8 provides ATRi resistance in both ATM-proficient and ATM-deficient cells. Cyclin C and CDK8 form part of the CDK8 kinase module of the RNAPII mediator complex, and deregulation of either gene has functional consequences for cancer development (59,60,80,81). We found that Cyclin C- or CDK8-deficient cells were selectively resistant to inhibitors of the RSR (ATR, CHK1 and WEE1) but did not function via regulation of CDC25A or re-establishment of the G2/M checkpoint (24). Instead, we discovered that depletion of Cyclin C (which is also destabilised in the absence of CDK8) reduced basal levels and ATRi- or CHK1i-induced DNA:RNA hybrids in S-phase. We predict that the reduction in DNA:RNA hybrids upon Cyclin C depletion reflects fewer collisions between transcription and replication machineries, which can cause genome instability through replication fork collapse and seDSB formation (9). Notably, eukaryotic transcription and replication are normally spatio-temporally co-ordinated in S-phase (82–85), and the location and regulation of DNA replication origins are also critical for limiting transcription-replication conflicts (7,72). By deregulating dormant-origin firing, ATRi or CHK1i are therefore likely to increase the probability of transcription-replication collisions and promote genome instability. Furthermore, abrogated ATR or CHK1 activity may exacerbate the DNA damage by increasing replication fork stalling (86) or by preventing effective replication fork stabilisation and restart, whereby ATR is activated at R-loop-stalled replication forks and is required for cell survival (43). Interestingly, we also identified that loss of factors such as YTHDF2 and RECQL5 increased ATRi sensitivity in our screens. These hits therefore support a general connection between the regulation of DNA:RNA hybrids and/or transcription-replication collisions and ATRi efficacy, since YTHDF2 regulates DNA:RNA hybrid stability to prevent their accumulation (87), and RECQL5 promotes the resolution of transcription-replication conflicts (73,74). Given that Cyclin C depleted cells had reduced basal levels of DNA:RNA hybrids, correlating with reduced ATRi/CHK1i-induced hybrid formation, it is possible that basal DNA:RNA hybrid levels could be used as a phenotypic biomarker in patient tumour samples to predict ATR and CHK1 inhibitor efficacy.

Correlating with reducing DNA:RNA hybrid formation, Cyclin C or CDK8 loss lowered ATRi- or CHK1i-induced replication stress. Consequently, we found that Cyclin C loss lowered ATRi-induced micronuclei formation, presumably as the reduction in replication stress was sufficient to limit under-replicated DNA from entering mitosis, which would be exacerbated upon G2/M checkpoint abrogation. On the other hand, CHK1 inhibition induced replication catastrophe, which was delayed in *CCNC* KO compared with WT cells. Notably, this delay was sufficient to promote cell survival in *CCNC* KO cells, emphasising how modulating the extent of replication stress in S-phase is sufficient to influence ATRi- and CHK1i-induced cell death. Importantly, we found that the impact of Cyclin C loss on replication stress and/or replication catastrophe could be phenocopied by pre-incubating cells with the transcription inhibitor DRB, but not by overexpressing RNase H1, therefore further highlighting a partial transcription-dependency of ATRi or CHK1i-induced replication stress that is alleviated by Cyclin C loss. Our data therefore highlight an important, and regulatable, role of transcription-associated replication stress that contributes to overall ATRi or CHK1i efficacy. We hypothesise that this is due to Cyclin C/CDK8 loss limiting transcription-replication conflicts, which otherwise increase upon inhibition of the RSR and potentiate genome instability and cell death. Correspondingly, Cyclin C loss did not rescue sensitivities towards aphidicolin or hydroxyurea, which cause replication stress yet reduce transcription-replication encounters (72), thereby supporting a specific role for Cyclin C loss in counteracting replication stress associated with transcription. Interestingly, although Cyclin C and CDK8 have largely been associated with transcriptional roles that could underpin this mechanism, both proteins have been recently implicated in DNA replication origin firing (88,89), which could contribute to the reduction in transcription-replication conflicts upon Cyclin C/CDK8 loss. Furthermore, although we did not observe a global reduction in RNAPII activity upon Cyclin C or CDK8 loss, local transcriptional control is now well documented in response to various DNA lesions including DSBs (76,90). The ATR homologue (Mec1) in budding yeast has also been reported to promote RNAPII degradation via INO80/PAF1 upon replication stress in order to limit transcription-replication conflicts (91). It is therefore intriguing to speculate that Cyclin C/CDK8 may provide an additional avenue of such regulation, for example through negative regulation by ATR to coordinate transcription with ongoing replication or replication stress. While such a model will require further investigation, we note that conserved ATM/ATR SQ phosphorylation motifs exist at the extreme C-terminus of Cyclin C and provide attractive targets for regulation by ATR.

In summary, our studies have provided a comprehensive overview of genes that, when inactivated, enhance or diminish ATRi efficacy, and highlighted how in some instances, but not others, these effects depend on functional ATM signalling. Further studies of various factors identified by our screens could facilitate successful stratification of cancer patients and/or elucidate new ATM-dependent pathways that compensate in the absence of functional ATR. Crucially, our results have highlighted the role of the RSR pathway in curtailing replication stress associated with transcription-replication encounters, and have shown that factors that either alleviate or enhance transcription-associated replication stress significantly influenced sensitivity to ATRi or CHK1i in ways that might in due course be exploitable in the clinical arena.

## DATA AVAILABILITY

RNA-seq: GEO accession number GSE165359 (https://www.ncbi.nlm.nih.gov/geo/query/acc.cgi?acc=GSE165359).

CRISPR–Cas9 screens: https://doi.org/10.5061/dryad.2v6wwpzm9.

Flow cytometry (https://flowrepository.org/): Repository IDs FR-FCM-Z3ES (Figure 4C), FR-FCM-Z3EM (Figure 6F, Supplementary Figure S8I), FR-FCM-Z3EX (Supplementary Figure S4A, Supplementary Figure S4C), FR-FCM-Z3EQ (Supplementary Figure S6D), FR-FCM-Z3NE (Supplementary Figure S9D).

## Supplementary Material

gkab628_Supplemental_Files

## Notes

Present address: Stephen P. Jackson, Wellcome/Cancer Research UK Gurdon Institute, University of Cambridge, Cambridge CB2 1QN, UK.
