# Supplementary material for: Loss of Cyclin C or CDK8 provides ATR inhibitor resistance by suppressing transcription-associated replication stress
Source: Nucleic Acids Res. 2021 Jul 30;49(15):8665–83. doi: 10.1093/nar/gkab628 (PMC8421211; doi:10.1093/nar/gkab628)
Supplement: gkab628_Supplemental_Files [file gkab628_supplemental_files.zip › Supplementary Information.pdf]

Supplementary information for

Loss of Cyclin C or CDK8 provides ATR inhibitor resistance by suppressing transcription-associated replication stress

Rebecca L. Lloyd, Vaclav Urban, Francisco Muñoz-Martínez, Iñigo Ayestaran, John C. Thomas, Christelle de Renty, Mark J. O'Connor, Josep V. Forment, Yaron Galanty and Stephen P. Jackson

Corresponding author

Stephen P Jackson

Email: s.jackson@gurdon.cam.ac.uk

This PDF file includes:

Supplementary Figures 1-9 and figure legends

Supplementary Table 1-4 legends

References for Supplementary Information

Supplementary Tables 1-4 and Supplementary Methods Tables are provided separately

Supplementary figures and figure legends:

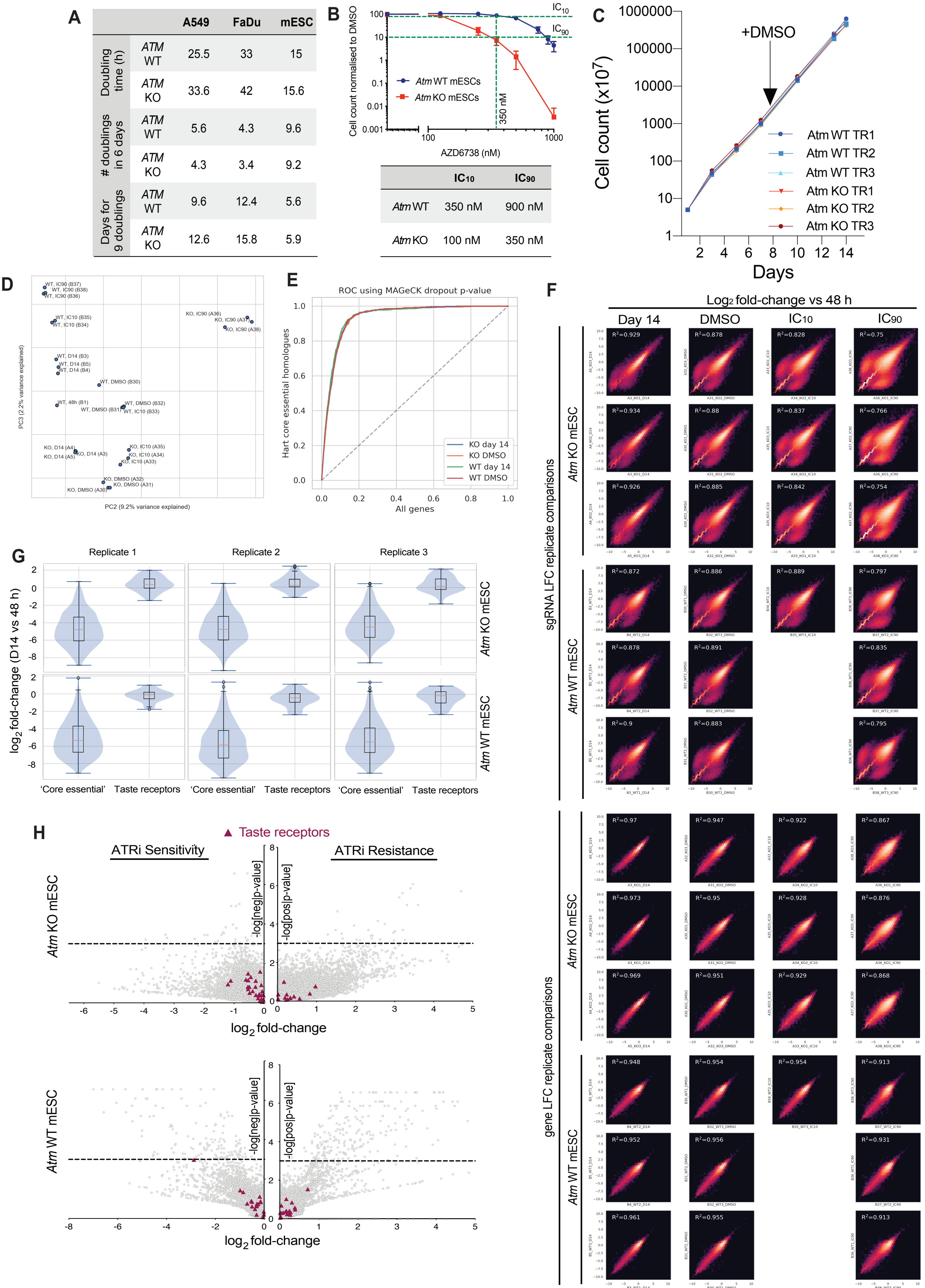

			A549	FaDu	mESC
Doubling time (h)	*ATM* WT		25.5	33	15
	ATM KO		33.6	42	15.6
# doublings in 6 days	*ATM* WT		5.6	4.3	9.6
	ATM KO		4.3	3.4	9.2
Days for 9 doublings	*ATM* WT		9.6	12.4	5.6
	ATM KO		12.6	15.8	5.9

	IC10	IC90
Atm WT	350 nM	900 nM
Atm KO	100 nM	350 nM

Supplementary Figure 1: Optimisation and quality control of CRISPR-Cas9 screens.

A) Doubling times including lag-phase for WT and ATM-deficient cells, calculated by counting cells during standard cell culture procedures.

B) Cell counts of *Atm* WT and KO mESCs after 6 days DMSO or AZD6738 treatment in screening format, used to calculate the respective IC_{10} and IC_{90} doses shown in the adjacent table. IC_{10} and IC_{90} doses are the inhibitory doses that resulted in a 10% or 90% reduction in cell count respectively. Error bars = mean ± S.D (biological n=2).

C) Cumulative cell counts of *Atm* WT and KO mESCs during the CRISPR-Cas9 screens, indicating comparable growth rates between the two genotypes. Cell counts are presented following the establishment of technical replicates (TR) until the end of DMSO treatment.

D) Principal component analysis using abundance normalised read counts, indicating that technical replicate 1 for IC_{10}-treated *Atm* WT mESCs (B33) clustered with DMSO-treated samples, and was thus excluded from subsequent analyses. Principal components 2 and 3 are shown. Principal component 1 primarily explained the impact of treatment in *Atm* KO mESCs and so is not shown.

E) Receiver operating characteristic (ROC) curves of MAGeCK p-values, discriminating between the mouse homologues of 'core essential' genes (1) and other genes in the library. Essential genes were identified in *Atm* WT or KO mESCs by comparing day 14 (pre-treatment) or DMSO-treated samples with the initial library representation at 48 h.

F) Scatter plots of Log_2 fold-changes for each sgRNA or gene, comparing individual replicates. Log_2 fold-changes were calculated by comparison to the initial library representation at 48 h, and R^2 values were calculated to indicate the strength of the correlation. The cloud of sgRNAs with negative Log_2 fold-changes are largely indicative of variations in low-read counts of statistically significant drop-outs.

G) Log_2 fold-changes for the mouse homologues of 'core essential' genes and genes encoding taste receptors, comparing day 14 and 48 h samples for each replicate and genotype, with taste receptors acting as negative controls.

H) Drop-out and enrichment MAGeCK analyses following AZD6738 treatment in *Atm* WT and KO mESCs, as shown in Figure 1B, highlighting genes encoding taste receptors (purple triangles) as negative controls. Statistically significant hits are above the dotted line (p-value <0.001), and taste receptors (negative controls) were not detected as hits in any of our screen outcomes (Supplementary Table 1).

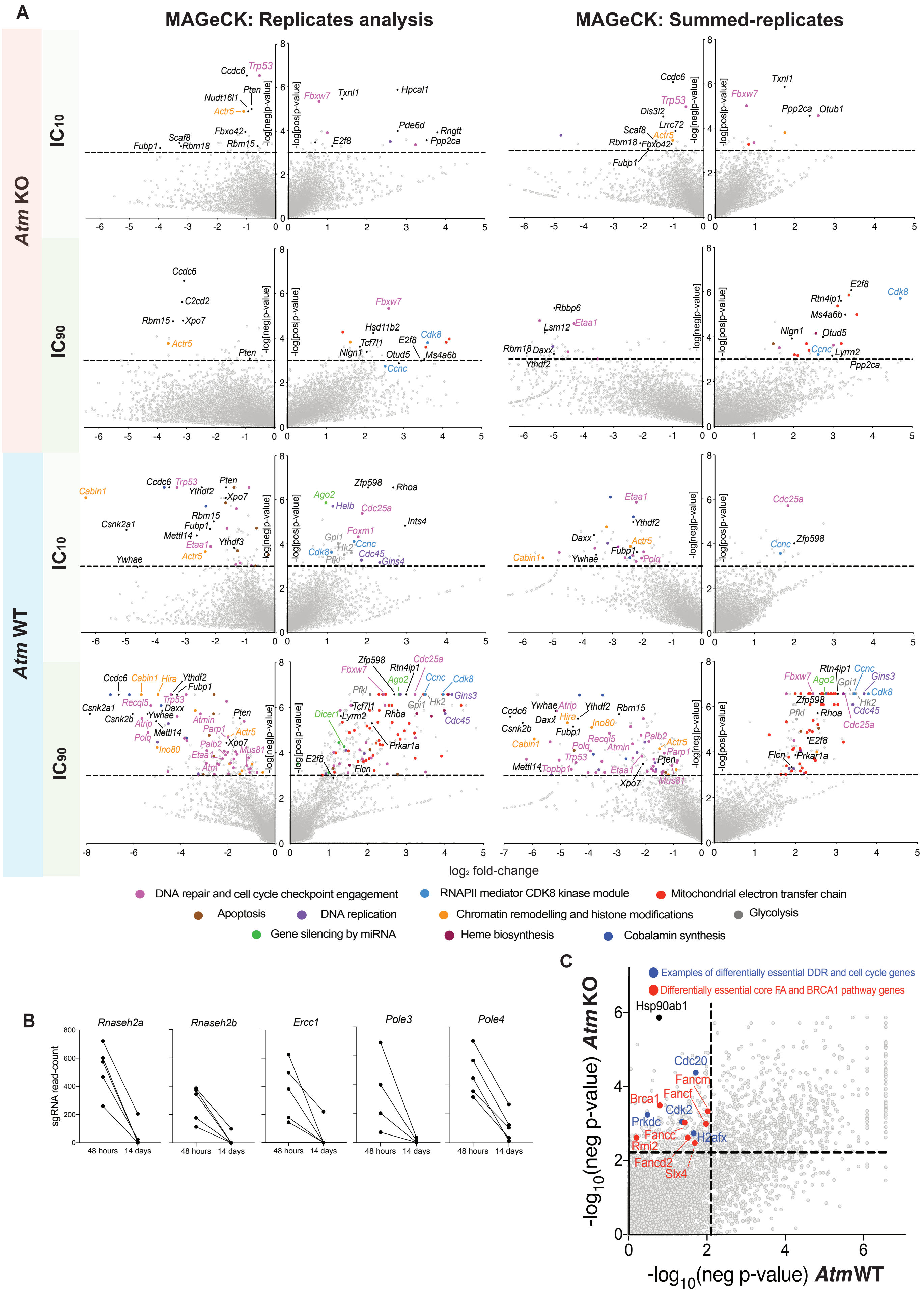
A
MAGeCK: Replicates analysis
MAGeCK: Summed-replicates
Atm KO
Atm WT
IC10
IC90
-log[neg|p-value]
-log[pos|p-value]
log2 fold-change
DNA repair and cell cycle checkpoint engagement
RNAPII mediator CDK8 kinase module
Mitochondrial electron transfer chain
Apoptosis
DNA replication
Chromatin remodelling and histone modifications
Glycolysis
Gene silencing by miRNA
Heme biosynthesis
Cobalamin synthesis
B
Rnaseh2a
Rnaseh2b
Ercc1
Pole3
Pole4
sgRNA read-count
48 hours
14 days
C
Examples of differentially essential DDR and cell cycle genes
Differentially essential core FA and BRCA1 pathway genes
Hsp90ab1
Cdc20
Fancm
Brca1
Fancf
Cdk2
Prkdc
Fancc
H2afx
Rmi2
Fancd2
Slx4
-log10(neg p-value) Atm KO
-log10(neg p-value) Atm WT

Supplementary Figure 2: CRISPR-Cas9 screen outcomes.

A) Drop-out and enrichment analyses following IC_{10} or IC_{90} AZD6738 treatments in *Atm* WT or KO mESCs. Genes were statistically ranked using MAGeCK analysis software following two approaches. 'Replicates analysis' used the built-in replicates function in MAGeCK, accounting for changes in read counts for all three replicates independently. For 'Summed-replicates', read counts for all three replicates were summed together prior to analysis. Top hits with a p-value <0.001 (dashed line) were classified into related pathways. The analyses which resulted in the greatest number of significant drop-out or enrichment hits (FDR <0.1) for each genotype are also shown in Figure 1B. Corresponding MAGeCK output files can be found in Supplementary Table 1.

B) Abundance-normalised sgRNA read counts for *Rnaseh2a, Rnaseh2b, Ercc1*, *Pole3* and *Pole4* in WT mESCs at 48 hours and 14 days (pre-treatment) post infection with the lentivirus sgRNA library. Read counts for day 14 samples are a mean of the 3 technical replicates. Each line represents a different sgRNA in the library.

C) Drop-out analyses comparing essential genes in *Atm* WT and KO mESCs, calculated between 48 h and day 14 (pre-treatment) samples. Genes were statistically ranked using MAGeCK analysis software. A selection of genes with an FDR < 0.1 in the *Atm* KO, but not WT, cells are labelled. Dashed lines indicate an FDR of 0.1.

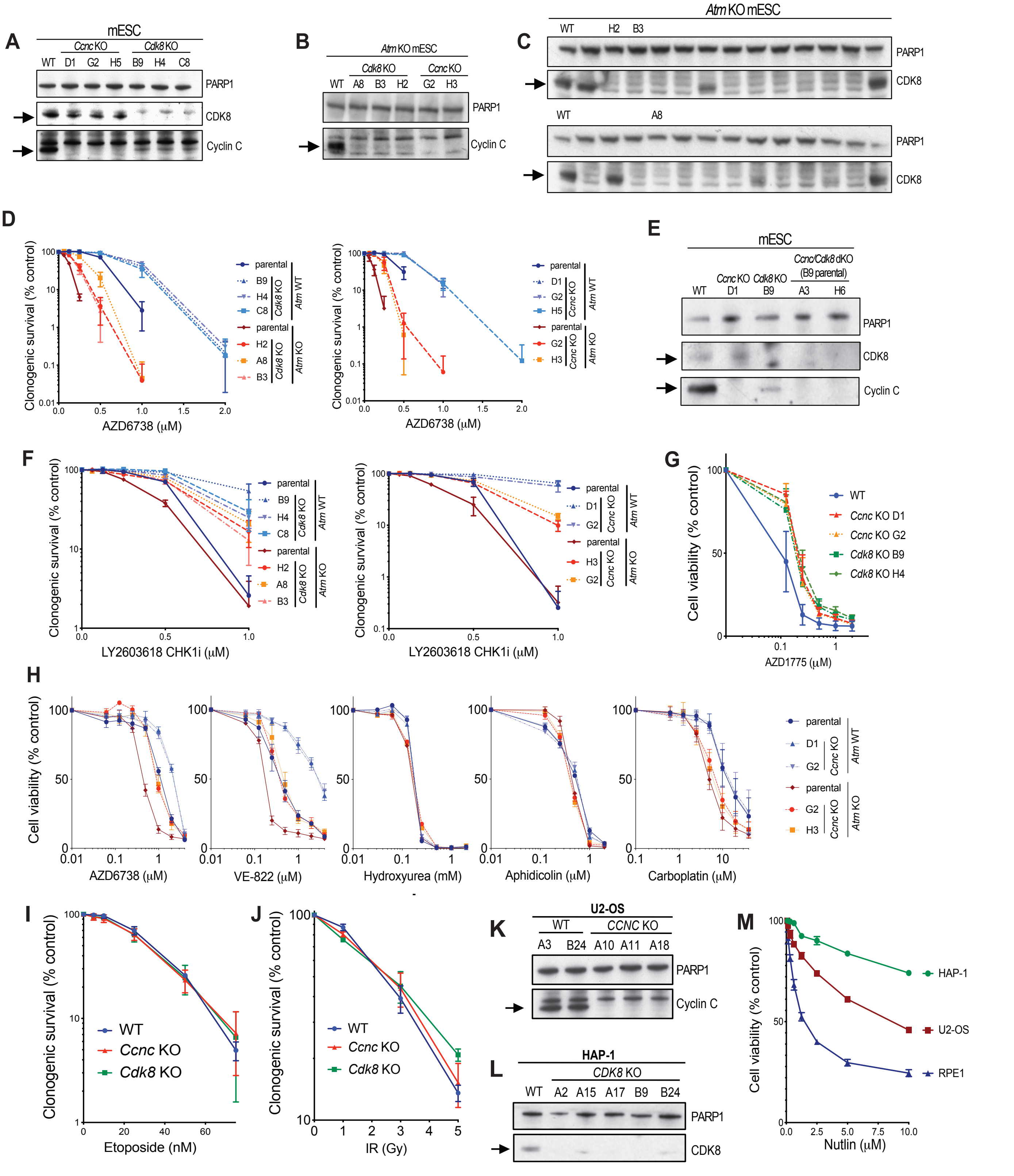

Supplementary Figure 3: Additional validation that Cyclin C/CDK8 loss selectively provides resistance to inhibitors of the RSR.

A) Immunoblots validating Cyclin C and CDK8 protein levels in *Atm* WT mESCs following CRISPR-mediated gene knockout.

B) Immunoblots of Cyclin C protein levels in *Atm* KO mESCs following CRISPR-mediated gene knockout of *Ccnc* or *Cdk8*.

C) Immunoblots screening for *Cdk8* knockout clones in *Atm* KO mESCs following CRISPR-mediated gene knockout. Clones used for further study are labelled.

D) Related to Figure 3A. Clonogenic survivals of *Ccnc*/*Cdk8* WT/KO *Atm* WT/KO mESCs treated with AZD6738. Error bars = mean ± S.D (biological n=3).

E) Immunoblots validating Cyclin C and CDK8 protein levels in single and double *Ccnc*/*Cdk8* KO mESCs following CRISPR-mediated gene knockout.

F) Related to Figure 3C. Clonogenic survivals of *Ccnc*/*Cdk8*WT/KO *Atm* WT/KO mESCs treated with the CHK1 inhibitor LY2603618. Error bars = mean ± S.D (biological n=3).

G) Results of MTT cell proliferation assays in *Ccnc/Cdk8* WT and KO mESCs after 4 days treatment with WEE1 inhibitor AZD1775. Error bars = mean ± S.E.M (biological n=3).

H) Results of MTT cell proliferation assays in *Ccnc* WT/KO *Atm* WT/KO mESCs after 4 days treatment with carboplatin, aphidicolin, hydroxyurea, or two independent ATR inhibitors (AZD6738, VE-822) which both inhibit ATR kinase activity in an ATP-competitive manner. Error bars = mean ± S.E.M (biological n=2-3).

I) Clonogenic survivals of WT and Ccnc/Cdk8 KO mESCs in response to etoposide treatment. Error bars = mean ± S.D (biological n=3).

J) Clonogenic survivals of WT and Ccnc/Cdk8 KO mESCs in response to IR treatment. Error bars = mean ± S.D (biological n=3).

K) Immunoblots validating Cyclin C protein levels in U2-OS cells following CRISPR-mediated gene knockout.

L) Immunoblots validating CDK8 protein levels in HAP-1 cells following CRISPR-mediated gene knockout.

M) Results of MTT cell proliferation assays in RPE-1, U2-OS and HAP-1 cells after treatment with nutlin, which prevents the negative regulation of p53 by MDM2. Error bars = mean ± S.D (n=3).

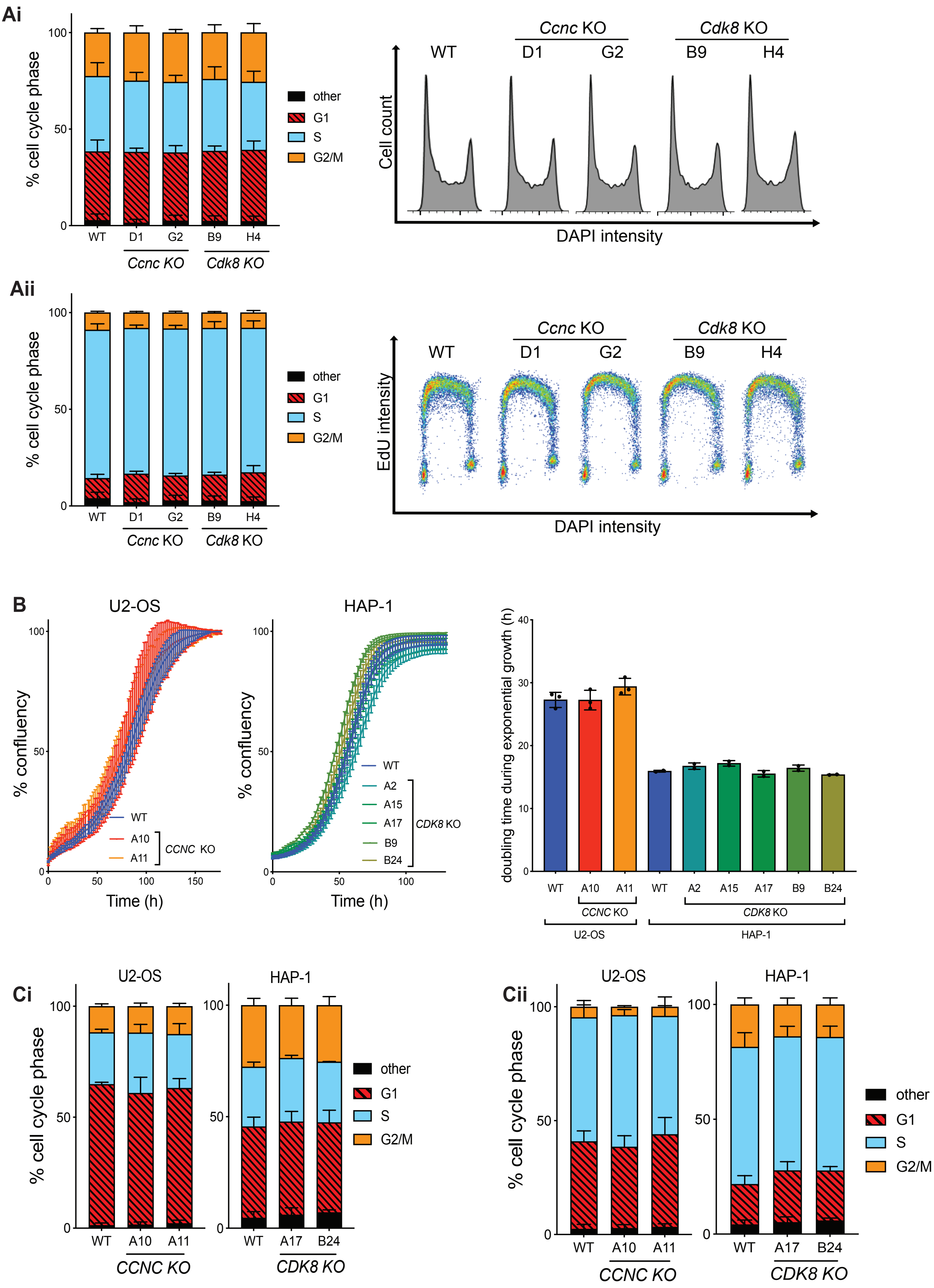
Ai
% cell cycle phase
WT
D1
G2
B9
H4
Ccnc KO
Cdk8 KO
other
G1
S
G2/M
Cell count
DAPI intensity
Aii
EdU intensity
B
U2-OS
HAP-1
% confluency
Time (h)
A10
A11
CCNC KO
A2
A15
A17
B24
CDK8 KO
doubling time during exponential growth (h)
Ci
Cii

Supplementary Figure 4: Loss of Cyclin C/CDK8 does not impact on cell cycle distributions or growth kinetics

A) Cell cycle distributions of *Ccnc/Cdk8* WT and KO mESCs assessed by i) DAPI histogram, and ii) EdU intensity versus DAPI. DNA content 2N = G1, 4N = G2/M. S phase cells are EdU positive following 30 min incubation with 10 μM EdU. Error bars = mean ± S.D (biological n=4).

B) Representative % confluencies of *CCNC* or *CDK8* U2-OS or HAP-1 WT and KO cells respectively over time. Error bars = mean ± S.D (technical n=5). % phase confluencies were calculated using Incucyte ZOOM 2018A software. Doubling times were calculated in GraphPad prism V.8 during the exponential phase of growth. Error bars = mean ± S.D (biological n=3 U2-OS, n=2 HAP-1).

C) Cell cycle distributions of *CCNC*/*CDK8* WT and KO U2-OS or HAP-1 cells respectively, assessed by i) DAPI histogram, and ii) EdU intensity versus DAPI. DNA content 2N = G1, 4N = G2/M. S phase cells are EdU positive following incubation with 10 μM EdU prior to fixation (20 min HAP-1, 45 min-1 h U2-OS). Error bars = mean ± S.D (biological n=3).

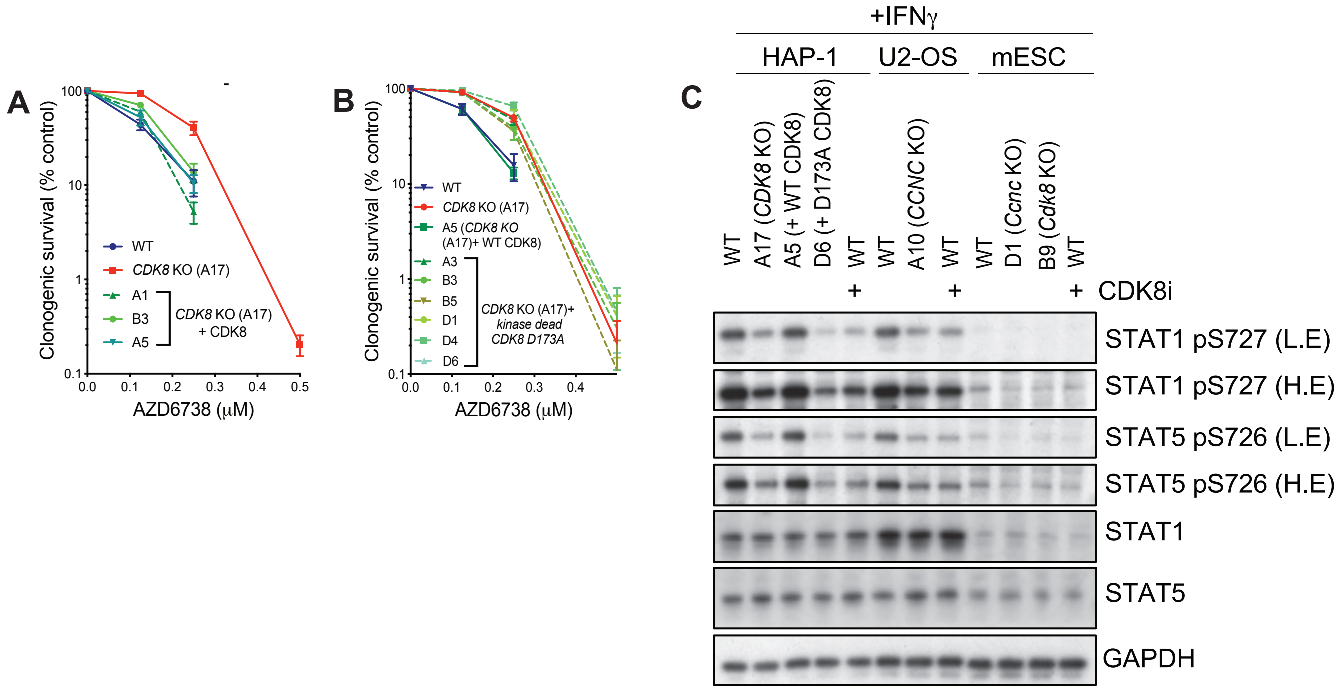

Supplementary Figure 5: CDK8 kinase activity is required for ATRi-treatment response

A) Related to Figure 3F. Clonogenic survivals of additional HAP-1 *CDK8* KO single-cell clones complemented with WT CDK8-GFP in response to AZD6738. Error bars = mean ± S.D (biological n=3).

B) Related to Figure 3F. Clonogenic survivals of additional HAP-1 *CDK8* KO single-cell clones complemented with kinase dead CDK8(D173A)-GFP in response to AZD6738. Error bars = mean ± S.D (biological n=3).

C) Immunoblots of CDK8 kinase targets STAT1 pSer-727 and STAT5 pSer-726 in Cyclin C or CDK8-deficient cells, including those complemented with WT or D173A kinase dead CDK8-GFP. Cells were pre-incubated with IFN-γ (2.5 ng/ml) for 4 h prior to sample collection. The CDK8 inhibitor BRD-6989 (15 μM, 24 h) was used as a positive control.

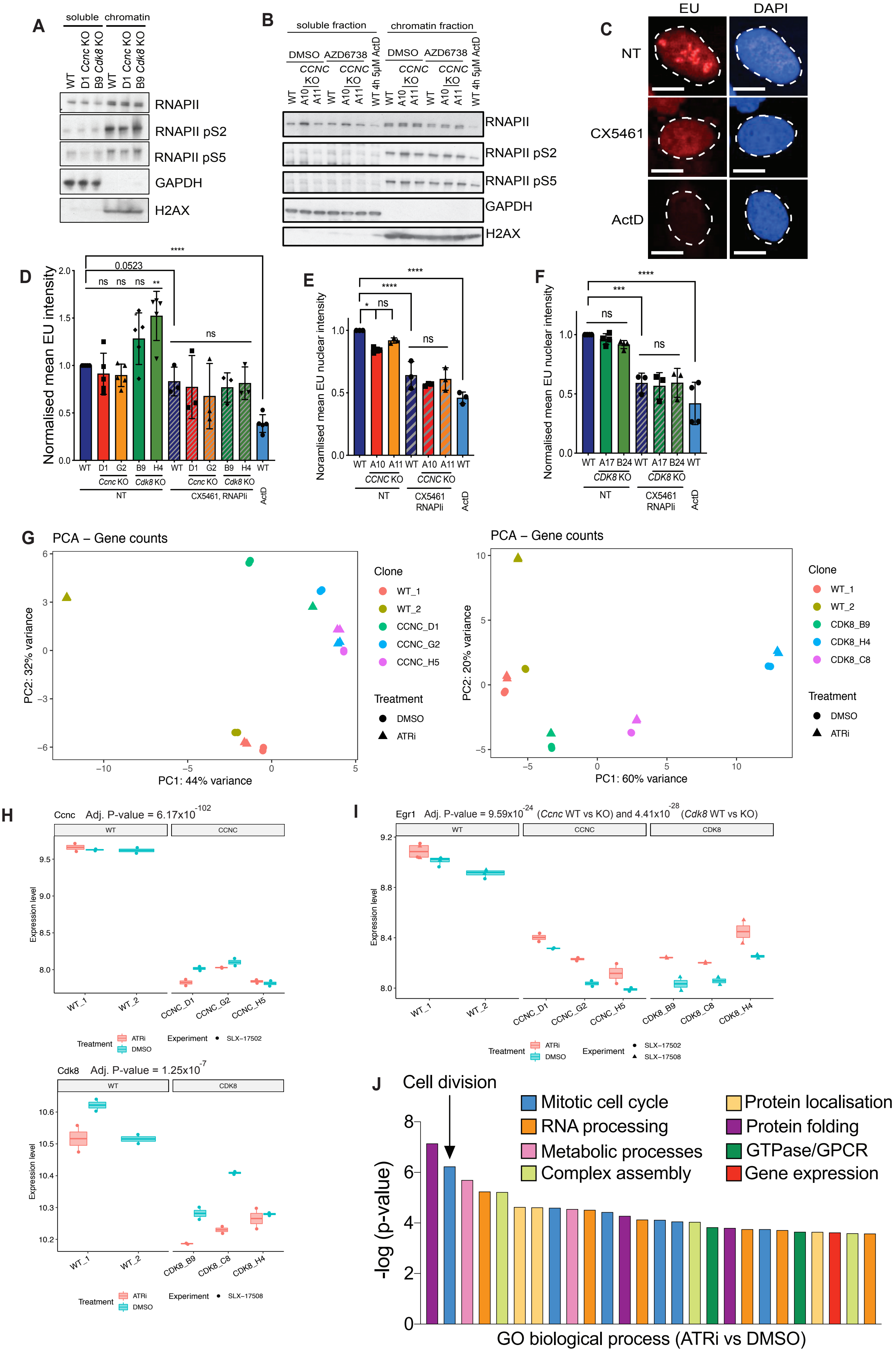
A
soluble
chromatin
WT
D1 Ccnc KO
B9 Cdk8 KO
RNAPII
RNAPII pS2
RNAPII pS5
GAPDH
H2AX
B
soluble fraction
chromatin fraction
DMSO
AZD6738
CCNC KO
WT
A10
A11
WT 4h 5μM ActD
RNAPII
RNAPII pS2
RNAPII pS5
GAPDH
H2AX
C
EU
DAPI
NT
CX5461
ActD
D
Normalised mean EU intensity
0.0523
****
ns
**
Ccnc KO
Cdk8 KO
NT
CX5461, RNAPli
ActD
E
Noramlised mean EU nuclear intensity
CCNC KO
NT
CX5461 RNAPli
ActD
F
Normalised mean EU nuclear intensity
***
CDK8 KO
NT
CX5461 RNAPli
ActD
G
PCA – Gene counts
PC1: 44% variance
PC2: 32% variance
Clone
WT_1
WT_2
CCNC_D1
CCNC_G2
CCNC_H5
Treatment
DMSO
ATRi
PCA – Gene counts
PC1: 60% variance
PC2: 20% variance
Clone
WT_1
WT_2
CDK8_B9
CDK8_H4
CDK8_C8
Treatment
DMSO
ATRi
H
Ccnc Adj. P-value = 6.17x10-102
Expression level
Treatment
ATRi
DMSO
Experiment
SLX-17502
Cdk8 Adj. P-value = 1.25x10-7
I
Egr1 Adj. P-value = 9.59x10-24 (Ccnc WT vs KO) and 4.41x10-28 (Cdk8 WT vs KO)
SLX-17508
J
Cell division
Mitotic cell cycle
RNA processing
Metabolic processes
Complex assembly
Protein localisation
Protein folding
GTPase/GPCR
Gene expression
-log (p-value)
GO biological process (ATRi vs DMSO)

Supplementary Figure 6: Assessing transcription in Cyclin C or CDK8 deficient cells.

A) Immunoblots of chromatinised RNAPII, and RNAPII CTD phosphorylation on Ser-2 and Ser-5 in WT and *Ccnc/Cdk8* KO mESCs.

B) Immunoblots of chromatinised RNAPII, and RNAPII CTD phosphorylation on Ser-2 and Ser-5 in WT and *CCNC* KO U2-OS cells. As indicated, cells were treated with DMSO or 1.5 μM AZD6738 for 24 h, or with 5 μM actinomycin D for 4 h prior to sample collection.

C) Representative images of U2-OS cells pre-treated with/without the RNAPI inhibitor CX5461 (3 h, 10 μM) or actinomycin D (3 h, 5 μM) prior to addition of 1 mM EU for 30 min. Brightest staining indicates nucleoli, as independently confirmed by co-localisation with fibrillarin. Scale bars = 12 μm.

D) Normalised mean EU intensities quantified by FACS in *Ccnc*/*Cdk8* WT and KO mESCs. Cells were incubated with 1 mM EU ± the RNAPI inhibitor CX5461 (10 μM) for 3 h prior to sample collection. EU intensities for each repeat were normalised to WT DMSO. Co-incubation of EU with 5 μM 3 h actinomycin D (ActD) was used as a positive control for total transcription inhibition. Error bars = mean ± S.D (biological n=5 non-treated and ActD, n=3 CX-5461).

E) Normalised EU intensities quantified by microscopy in *CCNC* WT and KO U2-OS cells. Cells were incubated for 3 h ± the RNAPI inhibitor CX5461 (10 μM), and 1 mM EU added 30 min prior to fixation. EU intensities for each repeat were normalised to WT DMSO. Co-incubation of EU with 5 μM 3 h actinomycin D (ActD) was used as a positive control for total transcription inhibition. Error bars = mean ± S.D (biological n=3).

F) Normalised mean EU intensities quantified by microscopy in *CDK8* WT and KO HAP-1 cells. Cells were incubated for 3 h ± the RNAPI inhibitor CX5461 (10 μM), and 1 mM EU added 30 min prior to fixation. EU intensities for each repeat were normalised to WT DMSO. Co-incubation of EU with 5 μM 3 h actinomycin D (ActD) was used as a positive control for total transcription inhibition. Error bars = mean ± S.D (biological n=4 non-treated, n=3 CX5461).

G) Principal component analysis of gene counts obtained from RNA-seq, with each graph generated using gene counts from the relevant multiplex. These data indicate that the AZD6738-treated WT biological replicate 2 samples deviated substantially from their expected behaviour and were thus excluded from subsequent analyses.

H) Expression levels of transcripts encoding Cyclin C or CDK8 in the respective WT and KO mESCs. Adjusted p-values were obtained by assessing treatment-independent changes in transcript levels between *Ccnc*/*Cdk8* WT and KO cells.

I) Expression levels of transcripts encoding EGR1 in WT, *Cdk8* KO or *Ccnc* KO mESCs, confirming reduced expression in *Ccnc* or *Cdk8* KO cells, consistent with at least two independent reports (2, 3). Expression levels were normalised between the two multiplexes. Adjusted p-values were obtained by assessing treatment-independent changes between *Ccnc*/Cdk8 WT and KO cells in the respective multiplex.

J) Gene Ontology analyses of genes upregulated upon ATRi treatment in a genotype-independent manner, highlighting that, as expected, genes involved in mitotic cell division were upregulated upon ATRi treatment, consistent with a previous report (4). Upregulated genes which had an adjusted p-value < 0.05 in both *Ccnc* WT/KO and *Cdk8* WT/KO analyses were classified by comparison to the *Mus musculus* reference, and the lead GO term for each cluster of processes is presented.

All Statistical analyses were performed using a one-way ANOVA test with multiple comparisons. P-values < 0.05 (*), 0.01 (**), 0.001 (***) and 0.0001 (****) were deemed statistically significant. In Supplementary Figure 6D, an independent ANOVA analysis was used to compare WT non-treated, CX-5461 and ActD-treated cells due to greater variance in the other samples.

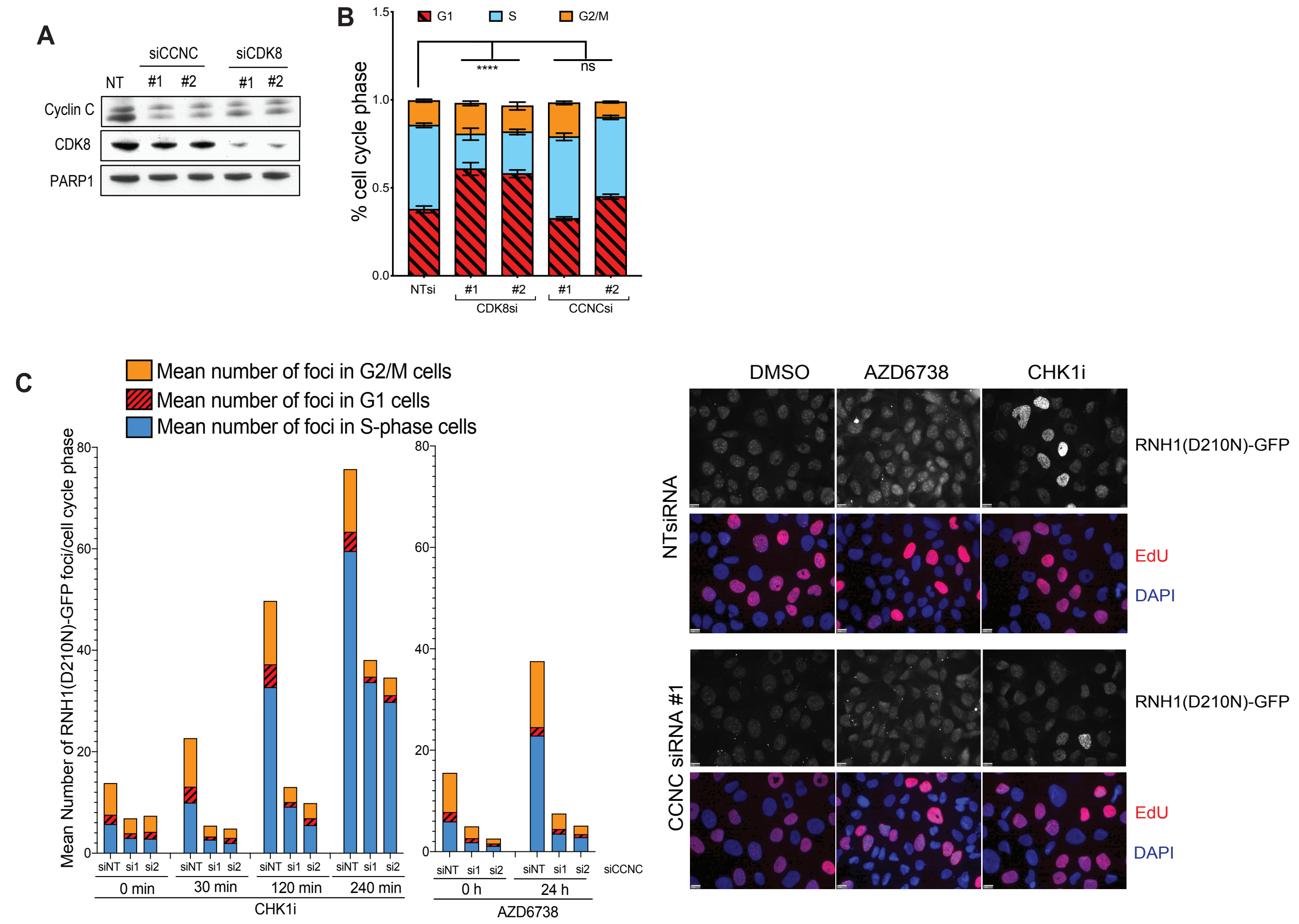

Supplementary Figure 7: Cyclin C loss reduces S-phase DNA:RNA hybrid formation

A) Immunoblots of Cyclin C and CDK8 levels 48 h after siRNA depletion in U2-OS cells.

B) Cell cycle distributions of U2-OS cells following siRNA-depletion of Cyclin C or CDK8 (n=4). Cell cycle distributions were determined from EdU and DAPI intensities calculated using fluorescence microscopy. G1 = EdU negative, 2N DNA content; S = EdU positive; G2/M = EdU negative, 4N DNA content. Statistical analyses were performed on % S-phase cells using a one-way ANOVA test with multiple comparisons. P-values < 0.05 (*), 0.01 (**), 0.001 (***) and 0.0001 (****) were deemed statistically significant.

C) Related to Figure 5A. Mean number of RNH1(D210N)-GFP foci/nuclei in G1, S and G2/M phases of the cell cycle in response to AZD6738 or CHK1i (LY2606368) treatment, following siRNA-depletion of Cyclin C. A representative repeat is provided, and the number of foci per cell cycle phase are plotted cumulatively. Cell cycle distributions were determined from EdU and DAPI intensities calculated using fluorescence microscopy. G1 = EdU negative, 2N DNA content; S = EdU positive; G2/M = EdU negative, 4N DNA content. Representative images are of RNH1(D210N)-GFP foci following treatment with DMSO, 24 h AZD6738 or 4 h CHK1i. Scale bar = 12 μm.

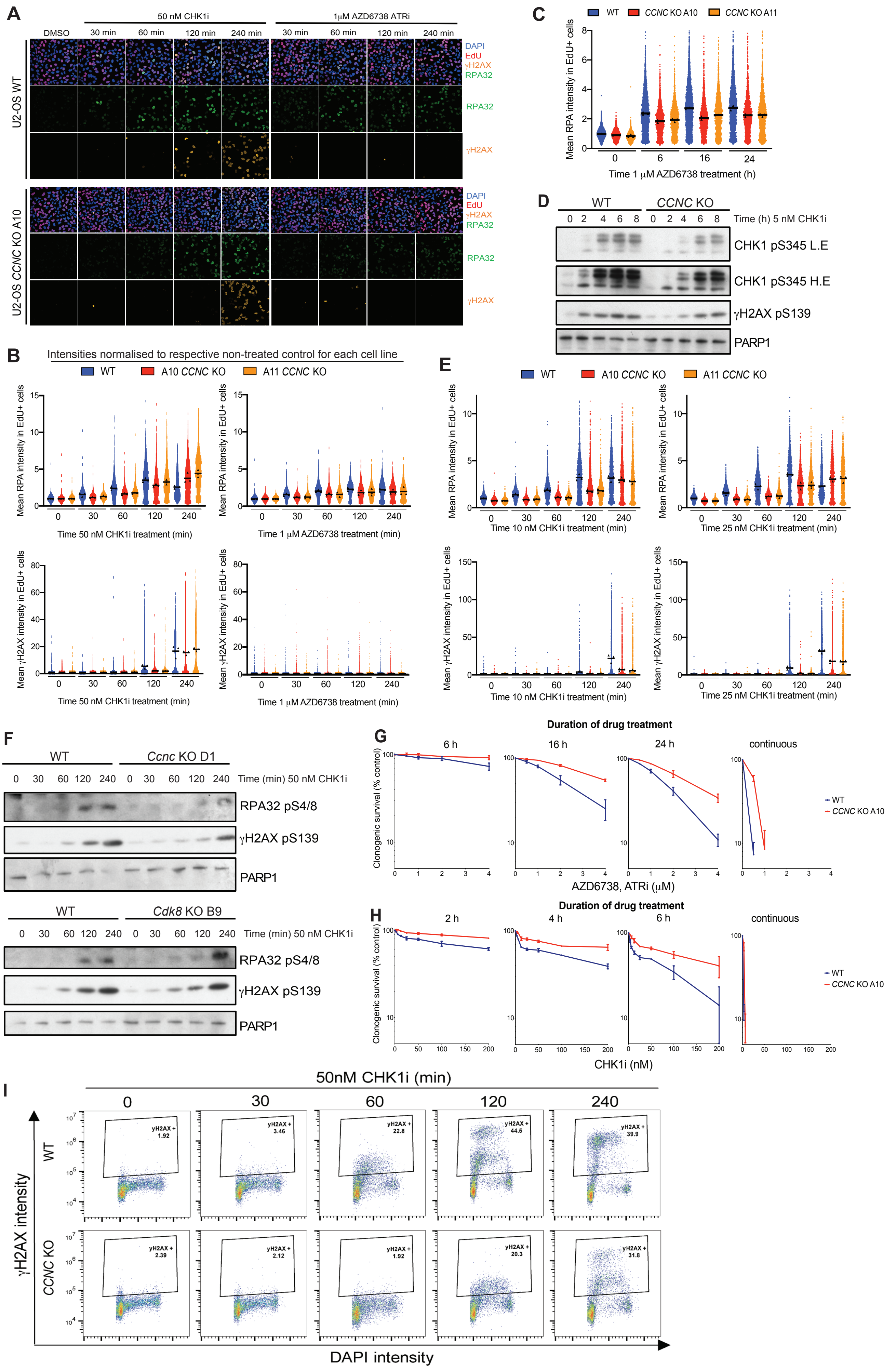
A
50 nM CHK1i
1μM AZD6738 ATRi
DMSO
30 min
60 min
120 min
240 min
U2-OS WT
U2-OS CCNC KO A10
DAPI
EdU
γH2AX
RPA32
B
Intensities normalised to respective non-treated control for each cell line
WT
A10 CCNC KO
A11 CCNC KO
Mean RPA intensity in EdU+ cells
Mean γH2AX intensity in EdU+ cells
Time 50 nM CHK1i treatment (min)
Time 1 μM AZD6738 treatment (min)
C
WT
CCNC KO A10
CCNC KO A11
Mean RPA intensity in EdU+ cells
Time 1 μM AZD6738 treatment (h)
D
WT
CCNC KO
Time (h) 5 nM CHK1i
CHK1 pS345 L.E
CHK1 pS345 H.E
γH2AX pS139
PARP1
E
WT
A10 CCNC KO
A11 CCNC KO
Time 10 nM CHK1i treatment (min)
Time 25 nM CHK1i treatment (min)
F
WT
Ccnc KO D1
Time (min) 50 nM CHK1i
RPA32 pS4/8
γH2AX pS139
PARP1
WT
Cdk8 KO B9
Time (min) 50 nM CHK1i
RPA32 pS4/8
γH2AX pS139
PARP1
G
Duration of drug treatment
6 h
16 h
24 h
continuous
Clonogenic survival (% control)
AZD6738, ATRi (μM)
WT
CCNC KO A10
H
Duration of drug treatment
2 h
4 h
6 h
continuous
Clonogenic survival (% control)
CHK1i (nM)
WT
CCNC KO A10
I
50nM CHK1i (min)
0
30
60
120
240
WT
CCNC KO
γH2AX intensity
DAPI intensity
yH2AX + 1.92
yH2AX + 3.46
yH2AX + 22.8
yH2AX + 44.5
yH2AX + 39.9
yH2AX + 2.39
yH2AX + 2.12
yH2AX + 1.92
yH2AX + 20.3
yH2AX + 31.8

Supplementary Figure 8: Loss of Cyclin C suppresses replication stress

A) Related to Figure 6B. Representative images were acquired at 40X magnification (field of view = 0.32 mm x 0.32 mm).

B) Related to Figure 6B. Mean chromatinised RPA32 and γH2AX intensities in EdU positive nuclei, following 0-240 min treatment with 50 nM CHK1i (LY2603638) or 1 μM AZD6738 (biological n=3). S-phase cells were labelled with 10 μM EdU for 30 min prior to pre-extraction and fixation. For each cell line, intensities were normalised to the non-treated sample of that cell line to account for basal differences. Mean intensities for each replicate are displayed as black triangles and were used to calculate the overall sample mean. Normalised mean intensities for each individual cell were combined across all three replicates and overlaid in blue (*CCNC* WT), red (*CCNC* KO A10) or orange (*CCNC* KO A11) for visualisation purposes of single-cell data.

C) Mean chromatinised RPA32 intensities in EdU positive nuclei normalised to non-treated WT cells, measured by immunofluorescence in *CCNC* WT and KO U2-OS cells, following 0, 6, 16 or 24 h treatment with 1 μM AZD6738 (biological n=3). S-phase cells were labelled with 10 μM EdU for 30 min prior to pre-extraction and fixation. Mean intensities for each replicate are displayed as black triangles and were used to calculate the overall sample mean. Mean intensities for each individual cell were normalised to the mean intensity of non-treated WT cells in each replicate, and all three replicates overlaid in blue (*CCNC* WT), red (*CCNC* KO A10) or orange (*CCNC* KO A11) for visualisation of single-cell data.

D) Immunoblots for markers of DSB formation, indicative of replication catastrophe. *CCNC* WT and KO U2-OS cells were treated for 0-8 h with 5 nM CHK1i (LY2603638) prior to lysis.

E) Mean chromatinised RPA32 and γH2AX intensities in EdU positive nuclei normalised to non-treated WT cells, measured by immunofluorescence in *CCNC* WT and KO U2-OS cells, following 0-240 min treatment with 10 and 25 nM CHK1i (LY2603638) (biological n=3). S-phase cells were labelled with 10 μM EdU for 30 min prior to pre-extraction and fixation. Mean intensities for each replicate are displayed as black triangles and were used to calculate the overall sample mean. Mean intensities for each individual cell were normalised to the mean intensity of non-treated WT cells in each replicate, and all three replicates overlaid in blue (*CCNC* WT), red (*CCNC* KO A10) or orange (*CCNC* KO A11) for visualisation of single-cell data.

F) Immunoblots for markers of DSB formation, indicative of replication catastrophe. *Ccnc* and *Cdk8* WT and KO mESCs were treated for 0-240 min with 50 nM CHK1i (LY2603638) prior to lysis.

G) Related to Figure 6D. Clonogenic survivals of *CCNC* KO U2-OS cells following short-term or continuous treatment with AZD6738. Error bars = mean ± S.E.M (biological n=3).

H) Related to Figure 6E. Clonogenic survivals of *CCNC* KO U2-OS cells following short-term or continuous treatment with CHK1i LY2603638. Error bars = mean ± S.E.M (biological n=3).

I) Related to Figure 6F. Representative FACS plots of γH2AX versus DAPI intensity after 0-240 min treatment of *CCNC* WT and KO U2-OS cells with 50 nM CHK1i (LY2603638). γH2AX positive cells were gated based on DMSO-treated WT cells.

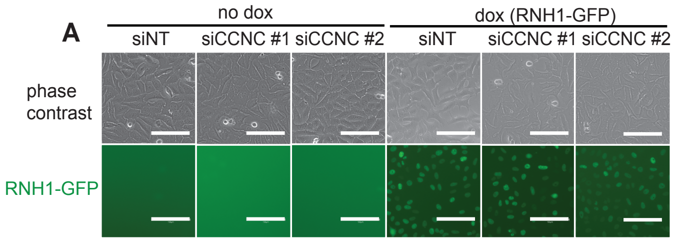

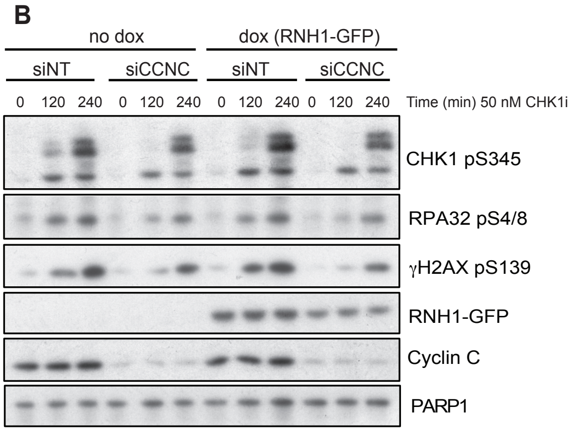

C

DRB treatment time (h)	Protocol stages for which this incubation time corresponds	Figure
0	no DRB pre or co-treatment	
1	1 h pre-incubation with DRB, prior to addition of ATRi/CHK1i	S9F
2	2 h total incubation time (1 h pre-incubation + 1 h co-treatment with ATRi/CHK1i)	S9F
	2 h pre-incubation with DRB, prior to addition of ATRi/CHK1i	7D-E
4	4 h total incubation time (2 h pre-incubation + 2 h co-treatment with ATRi/CHK1i)	7D-E

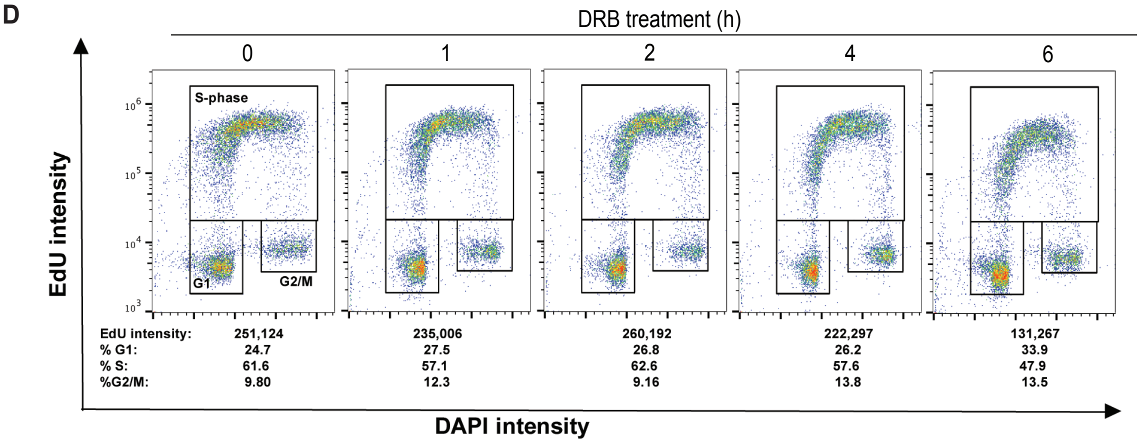

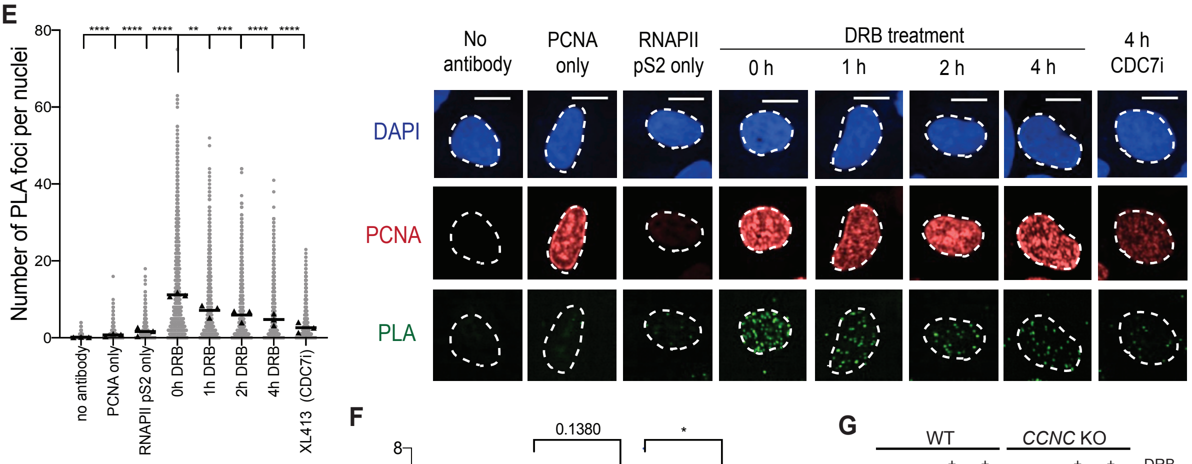

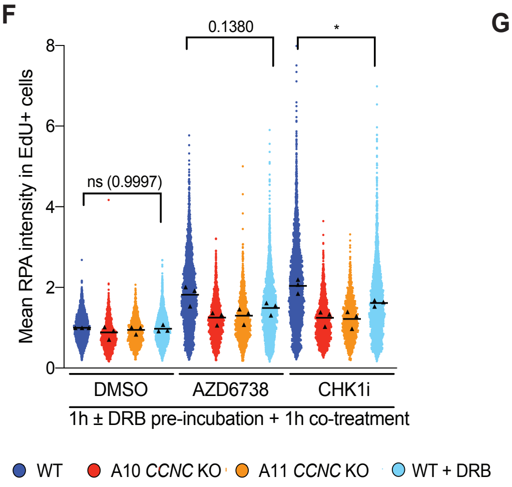

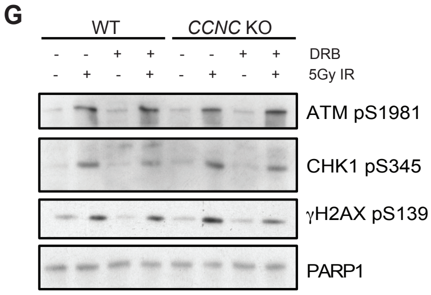

Supplementary Figure 9: Rescue of replication stress by Cyclin C loss is transcription dependent

A) Representative images of doxycycline-induced RNH1-GFP expression, indicating nuclear localisation, as confirmed for each biological replicate prior to performing the assays. Images were taken at 10X magnification using an EVOS M5000, 24 h after the addition of 1 ng/ml doxycycline. Scale bar = 150 μm.

B) Immunoblots for markers of DSB formation, indicative of replication catastrophe. U2-OS cells were siRNA-depleted of Cyclin C (siRNA #2), or transfected with the control NT siRNA, and RNH1-GFP expression induced by the addition of doxycycline 24 h prior to treatment with 50 nM CHK1i (LY2603638) for 0, 2 or 4 h.

C) Outline of how DRB treatment times corresponded to various protocols used to study the impact of transcription on ATRi/CHK1i-induced replication stress. The impact of these treatment times on the cell cycle and transcription-replication conflicts are shown in Supplementary Figure 9D and 9E respectively.

D) Cell cycle profiles of WT U2-OS cells treated with 100 μM DRB for the indicated time points assessed by EdU versus DAPI intensities using flow cytometry. DNA content 2N = G1, 4N = G2/M. S-phase cells are EdU positive following 30 min treatment with 10 μM EdU prior to fixation.

E) Mean number of PLA foci per nucleus assessing the co-localisation of RNAPII pSer-2 and PCNA (biological n=3). No antibody and single-antibody stainings were used as negative controls. WT U2-OS cells were treated with 5 μM CDC7i (XL413) for 4 h, or 100 μM DRB for the indicated time points prior to pre-extraction and fixation. Mean intensities for each replicate are displayed as black triangles and were used for the overall mean calculations and statistical analyses. Mean intensities for each individual cell were combined across all three replicates and overlaid for the visualisation of single-cell data. Statistical analyses were performed using one-way ANOVA with multiple comparisons. P-values < 0.05 (*), 0.01 (**), 0.001 (***) and 0.0001 (****) were deemed statistically significant. Representative images were taken using an Opera Phenix at 40X magnification, scale bars = 12 μm.

F) Mean chromatinised RPA32 intensities in EdU positive nuclei measured by immunofluorescence in *CCNC* WT and KO U2-OS cells, normalised to non-treated WT cells (biological n=3). Cells were pre-incubated with DMSO or 100 μM DRB for 1 h prior to 1 h co-treatment with DMSO, 1 μM AZD6738 or 50 nM CHK1i (LY2603638). This time point resulted in partial suppression of transcription-replication conflicts (Supplementary Figure 9E) but did not impact on DNA replication (Supplementary Figure 9D). S-phase cells were labelled with 10 μM EdU for 30 min prior to pre-extraction and fixation. Mean intensities for each replicate are displayed as black triangles and were used for the overall mean calculations and statistical analyses. Mean intensities for each individual cell were normalised to the mean intensity of non-treated

WT cells in each replicate, and all three replicates overlaid for visualisation of single-cell data. Statistical analyses were performed using one-way ANOVA analyses with multiple comparisons. P-values < 0.05 (*), 0.01 (**), 0.001 (***) and 0.0001 (****) were deemed statistically significant.

G) Immunoblots for markers of DSB formation. *CCNC* WT and KO U2-OS cells were pre-incubated with 100 μM DRB for 2 h prior to irradiation with 0 or 5 Gy. Cells were lysed 30 min after IR treatment.

Supplementary Table legends

Supplementary Table 1: MAGeCK analyses from CRISPR-Cas9 screens treating *Atm* WT and KO mESCs with AZD6738 (ATRi).

Results were generated using the software package MAGeCK (0.5.5) following exclusion of sgRNAs with low read counts from the DMSO control samples (5). WT IC_{10} technical replicate 1 was excluded from the analyses due to a close resemblance to the DMSO-treated samples (Supplementary Figure 1D). Given the data represent technical replicates (TR), data were analysed both by either summing the replicates (SUM <30ex), or by using the MAGeCK replicate function (REP <10ex) when comparing drug treatments. To identify drug drop-outs and enrichments, AZD6738 IC_{10} and IC_{90} treatments were independently compared to the relevant DMSO control. The analyses which provided the greatest number of significant hits (FDR <0.1) are visualised in Figure 1B-C, and all analyses visualised in Supplementary Figure 2A. To identify essential genes, samples were compared between Day 14 (pre-treatment) to 48 h.

Supplementary Table 2: Genotypes of *de novo* CRISPR-mediated gene KO cell lines.

Genotypes were identified using TIDe software and/or TOPO cloning.

Supplementary Table 3: Comprehensive list of drop-out and enrichment hits following AZD6738 treatment, identified in both *Atm* WT and *Atm* KO mESCs.

For each genotype, a comprehensive list of sensitisation and resistance hits was obtained by identifying genes which surpassed the significance threshold (p-value <0.001 or FDR <0.1, Supplementary Table 1) for at least one dose of AZD6738 (IC_{10} or IC_{90}), and when at least one analysis method was used. Hits which were identified in both *Atm* WT and KO mESCs are provided in this table.

Supplementary Table 4: RNA-seq analyses in *Ccnc* and *Cdk8* KO versus WT mESCs ± 4 hours AZD6738 treatment.

Lists of genes which were upregulated or downregulated in *Cdk8* and/or *Cdk8* KO mESC clones compared to WT control cells independent of treatment (ATRi independent), and genes which were up/downregulated upon AZD6738 treatment selectively in *Cdk8* and/or *Ccnc* KO mESCs, but not WT mESCs (ATRi + CCNC CDK8). Genes which were up/downregulated in both *Ccnc* and *Cdk8* KO mESCs are highlighted in green and classified by Gene Ontology analyses. For ATRi-independent genotype differences, significant hits had an adjusted p-value <0.05 and a fold-change <0.5 (downregulation) or >2 (upregulation). For ATRi-dependent genotype-specific differences, all hits with an adjusted p-value <0.05 are shown, as no genes surpassed a fold-change threshold <0.5 (downregulation) or >2 (upregulation). AZD6738-treated WT biological replicate 2 samples were excluded from the analyses after it showed substantial deviation from its expected behaviour when doing a principal component analysis (Supplementary Figure 6G) and other exploratory analyses (data not shown).

Supplementary Methods Table legends

1. Cell lines and their growth media
2. Antibodies
3. DsiRNA sequences
4. CrRNA sequences and associated primers for PCR amplification
5. RT-qPCR primers and amplicon information

References for Supplementary Information

1. Hart,T., Tong,A.H.Y., Chan,K., Van Leeuwen,J., Seetharaman,A., Aregger,M., Chandrashekhar,M., Hustedt,N., Seth,S., Noonan,A., *et al.* (2017) Evaluation and design of genome-wide CRISPR/SpCas9 knockout screens. *G3 Genes, Genomes, Genet.*, **7**, 2719–2727.
2. Chen,M., Liang,J., Ji,H., Yang,Z., Altilia,S., Hu,B., Schronce,A., McDermott,M.S.J., Schools,G.P., Lim,C.-U., *et al.* (2017) CDK8/19 Mediator kinases potentiate induction of transcription by NFκB. *Proc. Natl. Acad. Sci. U. S. A.*, **114**, 10208–10213.
3. Poss,Z.C., Ebmeier,C.C., Odell,A.T., Tangpeerachaikul,A., Lee,T., Pelish,H.E., Shair,M.D., Dowell,R.D., Old,W.M.

and Taatjes,D.J. (2016) Identification of Mediator kinase substrates in human cells using cortistatin A and quantitative phosphoproteomics. *Cell Rep.*, **15**, 436.

4. Saldivar,J.C., Hamperl,S., Bocek,M.J., Chung,M., Bass,T.E., Cisneros-Soberanis,F., Samejima,K., Xie,L., Paulson,J.R., Earnshaw,W.C., *et al.* (2018) An intrinsic S/G2 checkpoint enforced by ATR. *Science (80-.).*, **361**, 806–810.
5. Erard,N., Knott,S.R.V. and Hannon,G.J. (2017) A CRISPR Resource for Individual, Combinatorial, or Multiplexed Gene Knockout. *Mol. Cell*, **67**, 348-354.e4.
